# Molecular mechanisms of unique therapeutic potential of CUDC-907 for *MEF2D* fusion-driven BCP-ALL

**DOI:** 10.1038/s41392-025-02310-y

**Published:** 2025-07-23

**Authors:** Qing Xue, Ming Zhang, Yixiao Mo, Bo Jiao, Xuan Liu, Minghao Jiang, Yu Zhou, Yun Tan, Huimin Li, Jianming Zhang, Qianqian Zhang, Yunqi Li, Jianfeng Li, Xiaofang Ma, Duo-Hui Jing, Jian-Qing Mi, Jin Wang, Zhu Chen, Shu-Hong Shen, Sai-Juan Chen

**Affiliations:** 1 Shanghai Institute of Hematology, State Key Laboratory of Medical Genomics, National Research Center for Translational Medicine at Shanghai, Research Unit of Hematologic Malignancies Genomics and Translational Research of Chinese Academy of Medical Sciences, Ruijin Hospital, Shanghai Jiao Tong University School of Medicine, Shanghai, China; 2 Department of Hematology/Oncology, Shanghai Children’s Medical Center, School of Medicine, Shanghai Jiao Tong University, Shanghai, China

**Keywords:** Haematological cancer, Drug screening

## Abstract

*MEF2D* fusions are found in a special subtype of B-cell precursor acute lymphoblastic leukemia (BCP-ALL) with poor prognosis. In this study, we conducted high-throughput drug screenings using cell line and ex vivo cell model harboring, respectively, *MEF2D::HNRNPUL1(MH)* and *MEF2D::BCL9(MB)*, the two major *MEF2D* fusions. We identified CUDC-907 as a highly potent dual-target inhibitor of PI3K/HDAC, demonstrating remarkable efficacy in inducing robust lethality while maintaining selectivity for *MEF2D* fusion-expressing cells. CUDC-907 effectively induced apoptosis and promoted the down-regulation of pre-BCR signaling. We discovered that the hyperactivation of the PI3K-AKT signaling pathway, HDAC9, and BCL2 contributed to the sustained state of *MEF2D* fusion (+) BCP-ALL. Importantly, CUDC-907 exerted dual regulatory function by targeting the integrative pathways of MEF2D fusions. It suppressed the PI3K-CREB pathway and fusion gene expression, while simultaneously inhibited transcriptional activity regulated by the MEF2D fusion-HDAC axis. CUDC-907 demonstrated remarkable efficacy in patient samples carrying distinct *MEF2D* fusion variants in vitro. Furthermore, this compound’s effectiveness and safety were confirmed in both *MH/NRAS*^*G12D*^ BCP-ALL mouse model and *MB* patient-derived xenograft (PDX) model, outperforming conventional therapies. These results support the therapeutic potential of dual-pathway inhibition in MEF2D fusion (+) BCP-ALL and suggest CUDC-907 as a promising candidate for precision treatment in fusion-driven leukemias with similar molecular dependencies.

## Introduction

B-cell precursor acute lymphoblastic leukemia (BCP-ALL) is a heterogeneous hematological malignancy in which the lymphoblasts are blocked at distinct stages of immature B-lineage differentiation.^1^ Although major achievements have been obtained in the treatment of childhood BCP-ALL, with a 5-year overall survival (5-year OS) rate of 90%, the outcome of adult patients remains a challenge since the long-term survival rate is only around 50%.^1,2^ In the past decade, targeted therapies have led to improvements in the treatment of certain BCP-ALL subtypes, such as *BCR::ABL1* and Ph-like subtypes, especially when tyrosine kinase inhibitors are combined with hematopoietic stem cell transplantation (HSCT).^3–5^ Meanwhile, the next-generation sequencing technology has further refined molecular subtyping of BCP-ALL, and the newly identified disease drivers posit vulnerabilities for exploration of effective targeting agents. In this context, a subtype with *MEF2D* gene fused to multiple partners, accounting for about 5% of BCP-ALL, has drawn attention of hematology/oncology community.^1,2^ Characterized by clonal expansion of lymphoblasts at the pre-B cell stage, this subtype exhibits poor clinical prognosis, with 5-year OS rates of 33.3% in children and merely 15.6% in adults.^4^ Patients are generally unresponsive to conventional treatment regimens and HSCT.^6^ Therefore, there is an urgent need for effective therapeutic strategies against this BCP-ALL subtype.

Among the eleven *MEF2D* fusions so far identified,^6–10^ *MEF2D::BCL9 (MB)* and *MEF2D::HNRNPUL1 (MH)* are found in more than 80% of all cases.^7,8^ In fusion transcripts, the *MEF2D* portion consistently positions at the 5′ end, maintaining the DNA binding domain structure mediated by MADS and MEF2, while the 3′ moieties contain different partners.^6,11–13^ The N-terminal MADS and MEF2 domains are capable of promoting polymerization to exert the function of transcription factor (TF) and facilitating interactions with other cofactors, whereas the sequences in C-terminal partners might play a role in stabilizing the MEF2D fusion protein complex and empowering their aberrant functions.^9^ Of note, the RNA-seq data from fresh patient samples containing different *MEF2D* fusions exhibited highly similar transcriptomic landscape, indicating a typical phenocopy effect.^1,2^

It is known that pre-B cell receptor (BCR), a complex composed of two identical membrane-anchored immunoglobulin (Ig) μ heavy chains, two surrogate light chains subunits (λ5 and VpreB), and the signaling subunits Igα and Igβ, regulates pre-B cell proliferation and survival. In previous experiments, *MEF2D* fusions, especially *MH* and *MB*, have been shown to impede B cell development at the pre-B stage, upregulate the expression of genes involved in pre-BCR signaling, and provide survival advantage in *MEF2D* fusion-containing BCP-ALL cell lines.^6,10,11^ In a murine *MH* knock-in model, an early-stage B-cell development blockade has been noticed, which, in collaboration with *NRAS* mutation (*MH/NRAS*^*G12D*^), could develop towards a full-blown BCP-ALL. The differentially expressed genes (DEGs) upregulated in *MH* fusion mice were predominantly enriched in the MAPK signaling pathway, consistent with an enhanced pre-BCR signaling. Importantly, *Hdac9*, *Hdac4*, *Hdac1 and Bcl2* exhibited significantly higher expression levels in *MH* mice when compared to wild-type (WT) counterparts,^12^ in agreement with the earlier finding that patients with *MEF2D* fusions exhibited much higher *HDAC9* expression over other subtypes of BCP-ALL, whereas the transcriptional activity of *MEF2D* fusions towards *HDAC9* was stronger than that of WT *MEF2D*.^1^

Physiologically, the activated *HDAC9* would repress the expression of *MEF2D* via negative feedback.^9,13^ However, this circuitry has been disturbed by oncogenic *MEF2D* fusions. Chromatin immunoprecipitation (ChIP) experiments demonstrated an enhanced chromatin binding ability of MH to an array of target genes, including *HDAC9*.^1^ The overexpression of *HDAC9* has been shown to promote lymphoproliferative disorders and the development of lymphoma in Eμ-HDAC9 mouse models.^14^ Furthermore, our previous findings indicated that the higher affinity of MH fusion to the promoter of *HDAC9* could be facilitated by *HNRNPUL1* component in the protein complex.^12^ Overall, these observations underscore the critical role of *HDAC9* dysregulated by *MEF2D* fusions in driving leukemogenesis. Moreover, initial research on targeted treatment for *MEF2D* fusion BCP-ALL mainly focused on the high expression of HDAC9 using HDAC inhibitors.^7,12,15^ In the *MH/NRAS*^*G12D*^ leukemic model, the HDAC inhibitor Panobinostat, when combined with conventional chemotherapy, could effectively extend the survival of mice.^12^ These efforts, nevertheless, were constrained by relatively small scale of compound screenings and the limited diversity of screening model systems.^16,17^

In this study, we performed comparative studies at molecular, cellular, and organism levels of *MH* and *MB* fusions regarding their role in leukemogenesis and revealed key pathways shared by the two settings. Then, high-throughput drug screenings comprising 5512 compounds were conducted using a cell line harboring *MH* and an ex vivo cell model of *MB* fusion, respectively. We have identified CUDC-907, a potent dual inhibitor targeting both PI3K and HDAC, and currently under clinical trials for B cell lymphoma, as a promising therapeutic for *MEF2D* fusion-expressing BCP-ALL. CUDC-907 showed impressive effectiveness in selectively inducing apoptosis in *MEF2D* fusion-expressing cells, promoting the down-regulation of pre-BCR expression, and repressing leukemic cell proliferation. Its efficacy and safety were validated in in vivo models. Reversely, exploration of the mechanisms underlying the unique effects of CUDC-907 has enriched our understanding of the *MEF2D* fusions as disease drivers at the levels of pre-BCR signaling, transcriptional and epigenetic regulations, as well as intrinsic links between these key regulatory networks.

## Results

### Significant effects of inhibitors targeting PI3K signaling pathway and HDAC discovered by high-throughput drug screening for *MEF2D* fusion BCP-ALL cells

**Fig. 1:**
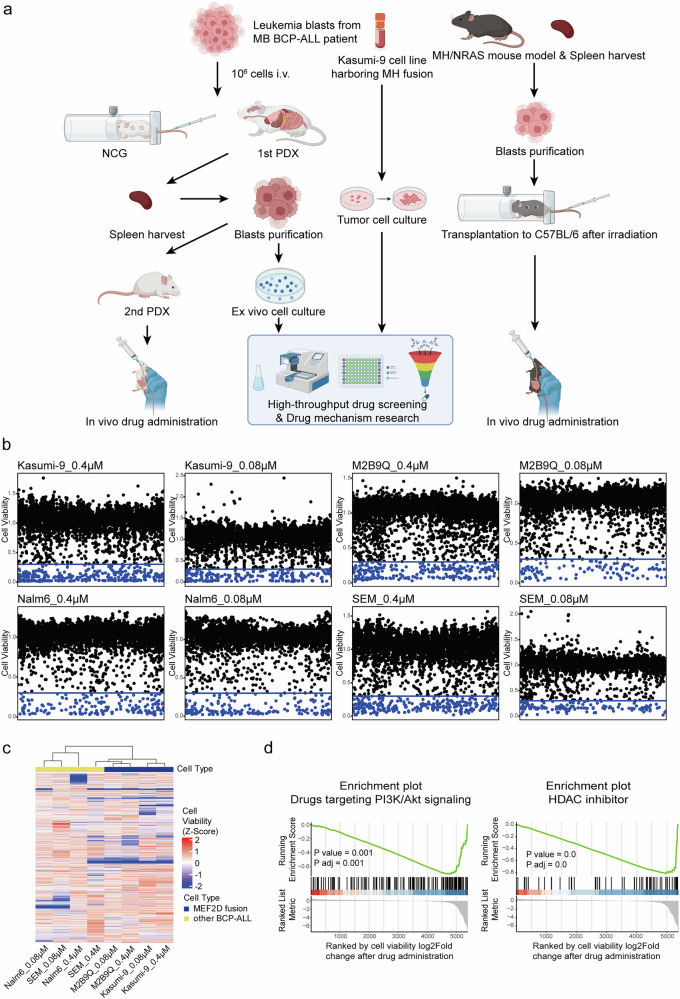
Establishment of in vitro and in vivo drug testing systems and emergence of PI3K and HDAC-targeting compounds as highly effective inhibitory agents against *MEF2D* fusion cells in large scale drug screening. **a** Schematic representation of the drug screening pipeline in *MEF2D* fusion cells, created with Biorender. **b** Dotplots of drug screening of different BCP-ALL cell lines. Non-MEF2D fusion cell lines Nalm6 and SEM served as controls. **c** Heatmap depicting the drug inhibition profiles of BCP-ALL cell lines. **d** Enrichment plot showing the effects of PI3K and HDAC-targeting drugs in the Kasumi-9 cell line. Drugs are ranked based on log2Fold change in cell viability after administration

The lack of stable *MB* fusion-dependent BCP-ALL cell lines has limited the study of leukemogenesis of *MB* fusion and development of novel therapies for *MEF2D* fusion (+) ALL. We therefore established a *MB* BCP-ALL ex vivo cell model named M2B9Q, derived from a primary sample of an 11-year-old pediatric BCP-ALL patient. The primary patient-derived xenograft (PDX) model was established by transplanting the patient’s leukemic blast cells into NOD/ShiLtJGpt-Prkdc^em26Cd52^Il2rg^em26Cd22^/Gpt (NCG) mice with severe functional deficiency of T, B, and NK cells. Spleen cells from the leukemic mice were harvested and purified for ex vivo culture (Fig. 1a left). In parallel, Kasumi-9 cell line derived from a BCP-ALL patient with *MH* was reported as a good model for in vitro molecular and cellular studies.^10,18^ These two *MEF2D* fusion BCP-ALL cell systems were well-suited for high throughput drug screening and investigation into pharmaceutical mechanisms. M2B9Q PDX model and our previously established *MH/NRAS*^*G12D*^ BCP-ALL mouse model^12^ (Fig. 1a right) were both useful for investigating the in vivo drug responses.

Besides, non-*MEF2D* fusion BCP-ALL cell lines Nalm6 and SEM were used as controls (Fig. 1b). Our chemical library consisted of 5512 small molecules covering wide range of known targets, including inhibitors of proteasome, kinases, and epigenetic modifiers, etc. We evaluated leukemia cell viability after 5 days of treatment at two different concentrations (0.4 μM and 0.08 μM) for each compound. The high-throughput drug screenings revealed similar inhibition profiles between *MEF2D* fusion cells and non-*MEF2D* fusion controls at a concentration of 0.4 μM. However, at a lower concentration of 0.08 μM, the pharmacological inhibition spectrum of *MEF2D* fusion cells differed significantly from those for controls, highlighting concentration-dependent selectivity of the identified compounds (Fig. 1c). We scrutinized the detailed cell viabilities in *MEF2D* fusion cells in the presence of 0.08 μM compounds, and those that could suppress cell viability below 30% were classified as “active compounds” (Supplementary Fig. 1a). In Kasumi-9 cell line, 307 active compounds were identified at a concentration of 0.4 μM, while 179 active compounds were detected at a lower concentration of 0.08 μM. Except for drugs targeting cell cycle, DNA damage/synthesis, and cytoskeleton drugs, the active compounds primarily targeted key signaling pathways and key nodes such as PI3K/AKT/mTOR pathway, histone deacetylase (HDAC), Nicotinamide Phosphoribosyl Transferase (NAMPT), P53, Bcl-2 family and MAPK signaling pathway (Supplementary Fig. 1b). In M2B9Q ex vivo cell model, 256 active compounds were observed at 0.4 μM and only 110 at 0.08 μM (Supplementary Fig. 1a). Consistently, the active compounds were categorized into P53 activators and inhibitors respectively against PI3K/AKT/mTOR signaling pathway, HDAC, NAMPT, Bcl-2 family, and MAPK signaling pathway (Supplementary Fig. 1c). We ranked the drugs of each target based on the log2FoldChange of cell viability after drug treatment and observed a significant enrichment of highly potent drugs in the PI3K signaling pathway and HDAC inhibitors (Fig. 1d).

### The dual PI3K/HDAC inhibitor CUDC-907 showing strong anti-*MEF2D* fusion BCP-ALL cell effect

**Fig. 2:**
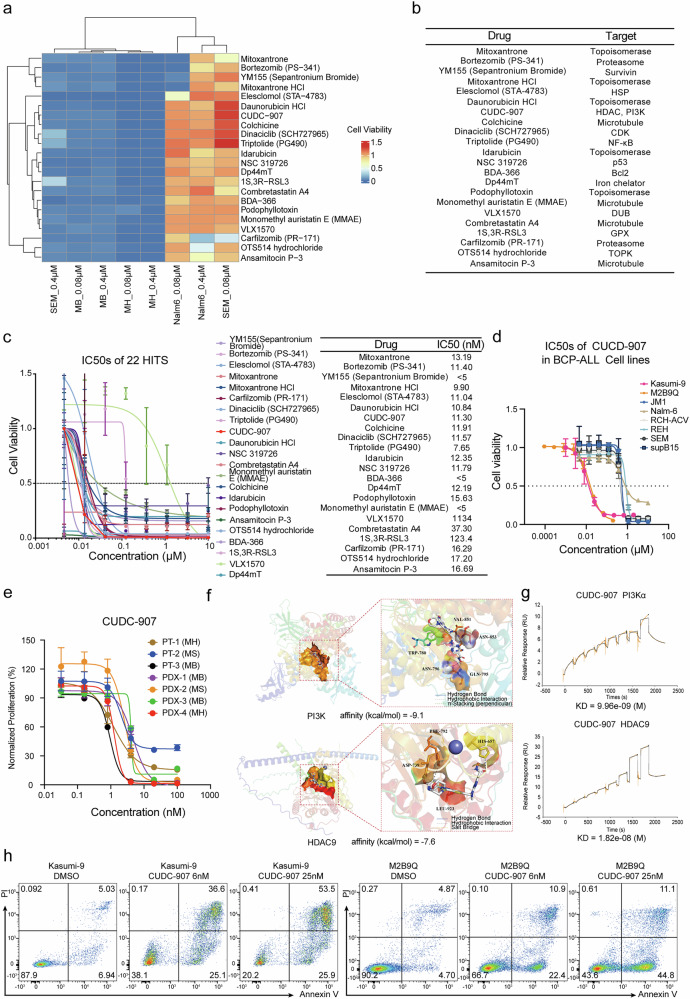
CUDC-907 Identified from 22 Hits with Induction of Leukemia Cell Apoptosis. **a** Heatmap depicting the effects of 22 hits in MEF2D fusion cells. **b** Targets of the 22 hits identified in MEF2D fusion cells. **c** IC50 values of the 22 hits in Kasumi-9 cell line. **d** IC50 values of CUDC-907 in BCP-ALL cell lines (*N* = 3). **e** The IC50 curves of CUDC-907 in patient primary samples and PDX cells. The fusion genes detected in the patients are indicated in parentheses. *MB* refers to *MEF2D::BCL9*, *MH* to *MEF2D::HNRNPUL1*, and *MS* to *MEF2D::SS18*. **f** The three-dimensional and two-dimensional interaction profiles between the ligand (CUDC-907) and PI3K/HDAC9 protein through molecular docking. **g** Surface plasmon resonance (SPR) analyses of recombinant human HDAC9 and PI3Kα protein affinities to CUDC-907. Yellow curves show the fitting to the raw data (shown in gray) using a 1:1 binding model. **h** Apoptosis assessed by annexin-V/propidium iodide (PI) staining in Kasumi-9 and M2B9Q cells treated with 6 nM or 25 nM CUDC-907 for 24 h, with corresponding DMSO controls

In drug screening for Kasumi-9 cells, there were 56 positive candidate compounds suppressing cell viability below 10% at both concentrations of 0.4 μM and 0.08 μM, whereas for M2B9Q cells, 28 positive candidates were identified (Supplementary Fig. 1a). A total of 22 hits overlapped in both *MH* and *MB* fusion BCP-ALL cells (Fig. 2a), of which, 11 targets for classical inhibitors against topoisomerase, microtubule and proteasome (Fig. 2b). Surprisingly, CUDC-907, through its two functional arms covering two targets PI3K and HDAC, resulted in an exceptionally potent pharmaceutical effect with a remarkably low IC50 value of only 11.3 nM in Kasumi-9 cells and 14.5 nM in M2B9Q ex vivo cell model (Fig. 2c and Supplementary Tables 1–2). Effectiveness of CUDC-907 in *MEF2D* fusion cells considerably surpassed that of other non-*MEF2D* fusion cell lines (IC50 value was 530.3 nM in JM1, 600.1 nM in Nalm6, 486.7 nM in RCH-ACV, 858.7 nM in REH, 465.4 nM in SEM, and 453.2 nM in sup-B15) and therefore exhibited high BCP-ALL targeting selectivity (Fig. 2d, Supplementary Fig. 1d and Supplementary Table 2). Furthermore, we collected three bone marrow (BM) blast samples from *MEF2D* fusion (+) BCP-ALL patients for drug sensitivity testing in vitro. Meanwhile, four blast samples of PDX mice constructed with cells from *MEF2D* fusion (+) BCP-ALL were also used in the same test. These seven samples also included a case with the third most common *MEF2D* fusion, *MEF2D::SS18*. CUDC-907 exhibited remarkable anti-proliferative effect across all samples, with an IC50 value ranging from 0.949 to 4.655 nM (Fig. 2e and Supplementary Table 3). Molecular docking analysis indicated that CUDC-907 inhibited PI3K activation by competing for the ATP-binding pocket of PI3K. In contrast, its binding to HDAC9 occurred via the conserved zinc ion-binding pocket that is characteristic of the HDAC protein family (Fig. 2f and Supplementary Fig. 1e, f). Surface plasmon resonance (SPR) experiments demonstrated that the compound exhibited strong binding affinity for PI3Kα and HDAC9, with dissociation equilibrium constants (KD) of 9.96 nM and 18.2 nM, respectively (Fig. 2g). Furthermore, CUDC-907 strongly induced apoptosis and an increasing proportion of leukemia cells in the G2/M phase (Fig. 2h and Supplementary Fig. 1g, h).

### Hyperactivation of HDAC and PI3K signaling pathways in *MEF2D*-fusion BCP-ALL cells

**Fig. 3:**
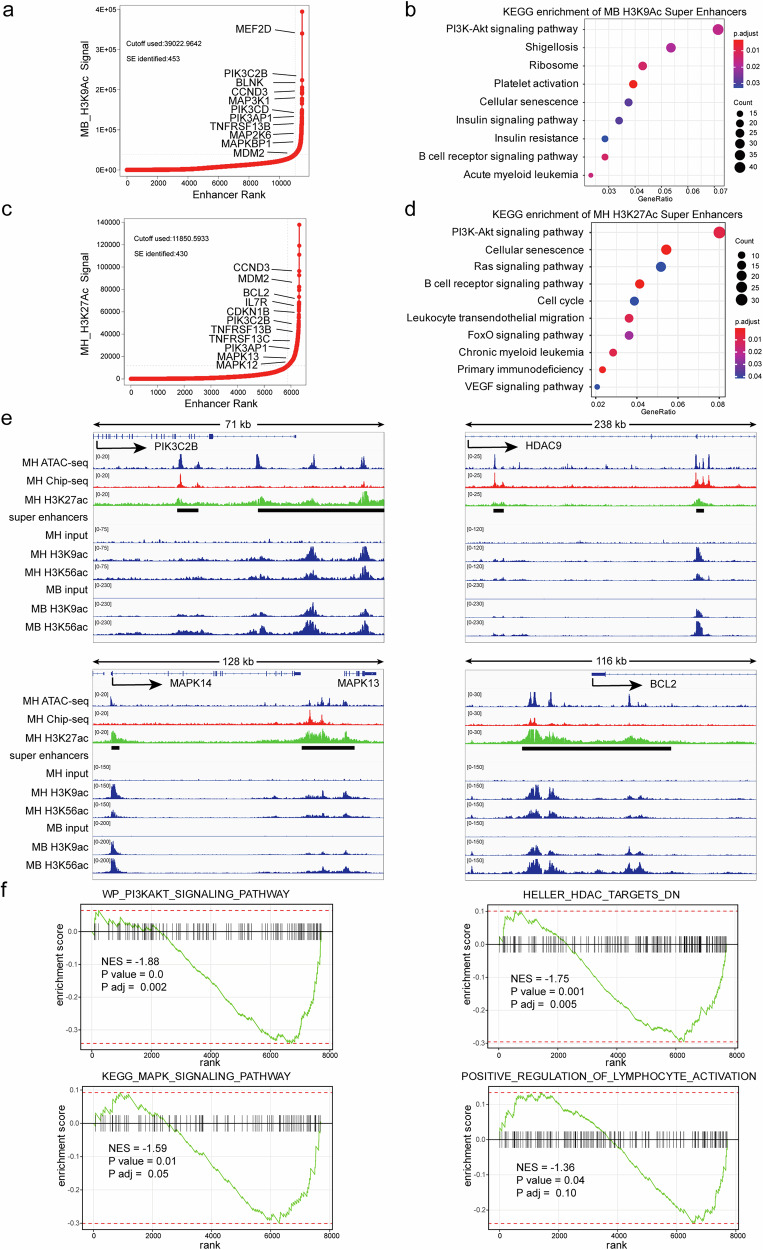
Hyperactivation of PI3K, HDAC, and MAPK Signaling Pathways in *MEF2D* Fusion BCP-ALL cells. **a** Profile of enhancers in M2B9Q cells based on H3K9ac ChIP-seq signal. Enhancers are ranked by signal intensity, with super-enhancers (SEs) highlighted in the top right quadrant. Genes associated with selected SEs are indicated. **b** Kyoto Encyclopedia of Genes and Genomes (KEGG) pathway analysis of genes associated with SEs involving MEF2D::BCL9 occupancy. **c** Profile of enhancers in Kasumi-7 cells based on H3K27ac ChIP-seq signal. Enhancers are ranked by signal intensity, with SEs highlighted in the top right quadrant. Genes associated with selected SEs are indicated. **d** KEGG pathway analysis of genes associated with SEs involving MEF2D::HNRNPUL1 occupancy. **e** Arrows under the top bars corresponding to genomic structures of representative genes indicate the locations and orientations of their transcription start sites. ATAC-seq, ChIP-seq, and H3K27ac signals in Kasumi-7, H3K9ac and H3K56ac signals in Kasumi-9 and M2B9Q, respectively, are shown. MH and MB SEs are denoted by black lines. Arrows indicate TSS locations and orientations. **f** Gene set enrichment analysis (GSEA) of differentially expressed genes (DEGs) between control and MEF2D::HNRNPUL1 knocked-down cells

The profound therapeutic efficacy observed upon inhibition of PI3K signaling pathway and HDAC prompted us to contemplate on the role played by these targets in pathogenesis. Since MH and MB function as aberrant TFs involving epigenetic regulatory machinery,^12,15^ we tried to address the major pathways contributing to leukemogenesis by using epigenetic analyses in *MB* and *MH* cell systems. Super enhancer (SE) is a cluster of regulatory elements that, through three-dimensional chromatin folding, comes into close proximity with its target gene and exhibits high levels of acetylation, driving robust and precise gene expression.^19^ After conducting a comprehensive analysis of H3K9Ac ChIP sequencing (ChIP-seq) on the M2B9Q ex vivo cell model, we identified a total of 453 SEs (Fig. 3a). Subsequent Kyoto Encyclopedia of Genes and Genomes (KEGG) pathway enrichment analysis revealed a significant enrichment of these SEs in the PI3K/AKT signaling pathway, which included genes such as *PIK3CB, BLNK, CCND3, PIK3CD, PIK3AP1*, and *MDM2* (Fig. 3a, b). Gene Ontology (GO) pathway enrichment analysis demonstrated these SEs were highly enriched in B cell activation pathways involving *TNFRSF13B* (Supplementary Fig. 2a and Fig. 3a). Several MAPK pathway genes including *MAP3K1, MAP2K6* and *MAPKBP1* were also classified as SEs (Fig. 3a). We then re-analyzed the previously published experimental data of H3K27Ac ChIP-seq from the Kasumi-7 cell line, another cell model expressing *MH*,^10^ and identified 430 SEs (Fig. 3c). KEGG pathway enrichment analysis showed significant enrichment in the PI3K/AKT signaling pathway as well, including *CCND3*, *MDM2*, *BCL2*, *IL7R*, *CDKN1B*, *PIK3C2B*, and *PIK3AP* (Fig. 3c, d). GO pathway analysis indicated high enrichment of these SEs in B cell activation pathways involving *TNFRSF13B* and *TNFRSF13C* (Supplementary Fig. 2b and Fig. 3c). MAPK pathway genes such as *MAPK13* and *MAPK12* were also classified as SEs (Fig. 3c). Notably, 175 genes overlapped in SEs between *MB* and *MH* cell systems, including *MEF2D, CCND3, MDM2, BLNK, IGLL1, VPREB1, CD79B, TNFRSF13B, PIK3C2B*, and *PIK3CD*. The corresponding Assay for Transposase-Accessible Chromatin sequencing (ATAC-seq) and ChIP-seq data of Kasumi-7 cells revealed open chromatin regions and MH binding peaks for *PIK3C2B*, *HDAC9*, *MAPK13*, and *BCL2* genes, indicating transcriptional activation by *MH* fusion. Notably, activation of this four-gene set was also observed in *MB* cells (Fig. 3e, under the top bar representing genomic structure of the gene, upper three bars in each panel). In the acetylation sequencing of H3K9 and H3K56 in Kasumi-9 and M2B9Q cells, we observed the same target genes activation (Fig. 3e, middle and lower three bars, respectively, in each panel). On the contrary, when *MH* fusion gene was knocked down in Kasumi-7 cells and leukemia cell death was induced as previously described,^10^ our re-analysis of relevant data found PI3K/AKT signaling pathway, HDAC targets, MAPK signaling pathway, and B cell activation pathways were significantly down-regulated (Fig. 3f). Therefore, the two common *MEF2D* fusion BCP-ALL subtype variants should share similarity in terms of hyperactivated PI3K/AKT, HDAC, MAPK signaling pathways, and BCL2 family. Meanwhile, re-analysis of previous RNA-seq data^10,12^ showed that overexpression of the *MH* fusion gene in REH cells downregulated the lymphocyte differentiation pathway. In contrast, knockdown of *MH* in Kasumi-9 cells upregulated this pathway and promoted lymphocyte differentiation through modulation of shared key genes (Supplementary Fig. 2c–e). In addition, both *MH* and *MB* SEs were enriched for BCR signaling pathway (Fig. 3b, d). Of note, *MEF2D* fusion (+) leukemia blasts did not develop into immature B cells and therefore had no expression of the whole set of BCR signaling molecules. In fact, the absence of the term “pre-BCR” in GO/KEGG databases obscured the assignment of some common genes that actually belong to the pre-BCR pathway, such as *VPREB1*, *IGLL1*, *ZAP70*, *CD79A*, and *CD79B*. More importantly, the PI3K signaling pathway has been shown to serve as the central downstream effector of the pre-BCR signaling pathway, and restoration of PI3K signaling pathway activation can rescue the loss of tonic pre-BCR signaling.^20,21^ Taken together, PI3K pathway involvement should be considered one of the characteristics for the pre-B stage of *MEF2D* fusions of BCP-ALL.

### Inactivation of both PI3K-CREB-MEF2D pathway and MEF2D-HDAC axis by CUDC-907

**Fig. 4:**
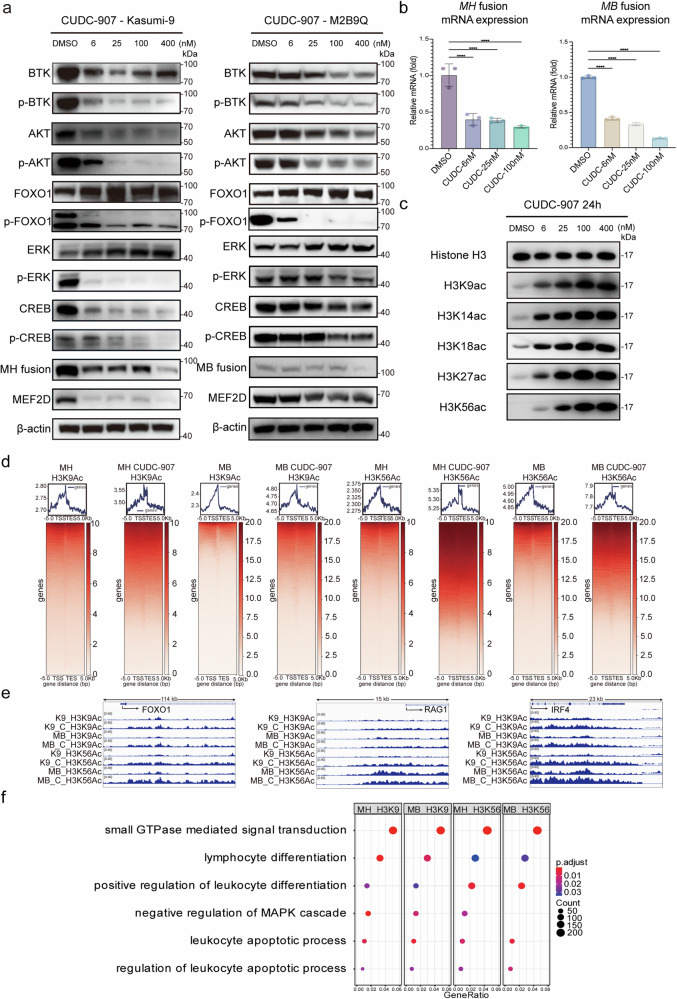
CUDC-907: Inhibition of the PI3K/AKT Pathway and Increased Histone H3 Acetylation. **a** Immunoblot analysis of PI3K signaling pathway downstream components in lysates from Kasumi-9 or M2B9Q cells treated with DMSO or various concentrations of CUDC-907 for 24 h, as indicated. β-actin served as a loading control. **b** mRNA expression of MH and MB fusions measured by qRT-PCR in Kasumi-9 and M2B9Q cells treated with DMSO or 25 nM CUDC-907 for 24 h (*N* = 3). **c** Immunoblot analysis of H3 histone acetylation in Kasumi-9 cells treated with CUDC-907 for 24 h. **d** ChIP-seq analysis of H3K9Ac and H3K56Ac in Kasumi-9 cell line and M2B9Q ex vivo cell model with or without CUDC-907 treatment for 24 h. Genome-wide heatmaps of H3K9Ac and H3K56Ac are shown. **e** Profiles at FOXO1, RAG1, and IRF4 gene loci in ChIP-seq data from Kasumi-9 cell line and M2B9Q ex vivo cell model treated with or without CUDC-907 for 24 h. K9 denotes the Kasumi-9 cell line, while MB refers to the M2B9Q ex vivo cell model. C indicates the CUDC-907 treatment group, and Ac signifies histone acetylation. Specifically, K9_C_H3K9Ac represents the acetylation of histone H3K9 in the Kasumi-9 cell line following CUDC-907 treatment, with similar notations applying to other related conditions. **f** Cluster Gene Ontology (GO) enrichment profiles of elevated acetylation of genes in Kasumi-9 cell line and M2B9Q ex vivo cell model after CUDC-907 treatment

As a PI3K inhibitor, CUDC-907 effectively suppressed the expression and phosphorylation of BTK and AKT, which play important roles in triggering clonal expansion of pre-B cells (Fig. 4a). The FOXO1 TF, a major downstream effector of the PI3K/AKT pathway, is negatively regulated through AKT-mediated phosphorylation and inactivation of FOXO1 has been implicated in the development of pre-B ALL.^22–24^ Following the treatment of CUDC-907, the decrease in AKT expression and phosphorylation resulting in an upregulation of FOXO1 expression and reactivation of its target genes related to pre-B cell development program (Supplementary Fig. 3a, b). The significance of the PI3K/MAPK pathway in pre-BCR proliferation and differentiation has long been recognized and attributed to the ERK1/2 cascade.^25,26^ CUDC-907 upregulated the protein level of ERK, but caused a decrease in ERK phosphorylation (Fig. 4a). Consequently, there was a reduction observed in the expression and phosphorylation of CREB, which acts as a downstream mediator of ERK and facilitates the transcriptional activity of MEF2D. Knockdown of CREB has been shown to reduce *MEF2D* fusion TF expression.^10,27^ Similarly, the mRNA levels of *MEF2D* fusions were significantly decreased after CUDC-907 treatment (Fig. 4b). As a result, both of MEF2D and MEF2D fusion proteins were diminished (Fig. 4a). Meanwhile, the aberrant fusion of MEF2D disrupts its canonical transcriptional regulation, which is typically repressed by its target gene *HDAC9* expression in normal tissues.^9,13^ CUDC-907, a pan-HDAC inhibitor, effectively inhibited various members of the HDAC family, including HDAC9 (Supplementary Fig. 3c).

The complex formed by MEF2D, HDAC3, along with HDAC4, regulates these processes, with HDAC3 primarily mediating MEF2D acetylation.^28^ Treatment with CUDC-907 significantly increased acetylation at various H3 histone residues (Fig. 4c). ChIP-seq analysis of Kasumi-9 and M2B9Q cells upon effect of CUDC-907 confirmed a concurrent elevation in histone H3 acetylation at lysine residue 9 and 56, revealing a substantial enhancement in binding peaks (Fig. 4d). Consistent with previous findings, CUDC-907 treatment activated FOXO1 and its target genes, including *IRF4* and *RAG1* (Fig. 4e). Genes with elevated histone H3 acetylation levels were mainly enriched in small GTPase mediated signal transduction, lymphocyte differentiation, negative regulation of MAPK cascade, leukocyte apoptotic process, all of which were closely associated with leukocyte differentiation and apoptosis (Fig. 4f).

### RNA-seq analysis confirmed effective upregulation of apoptotic pathway and downregulation of pre-BCR signaling pathway by CUDC-907

**Fig. 5:**
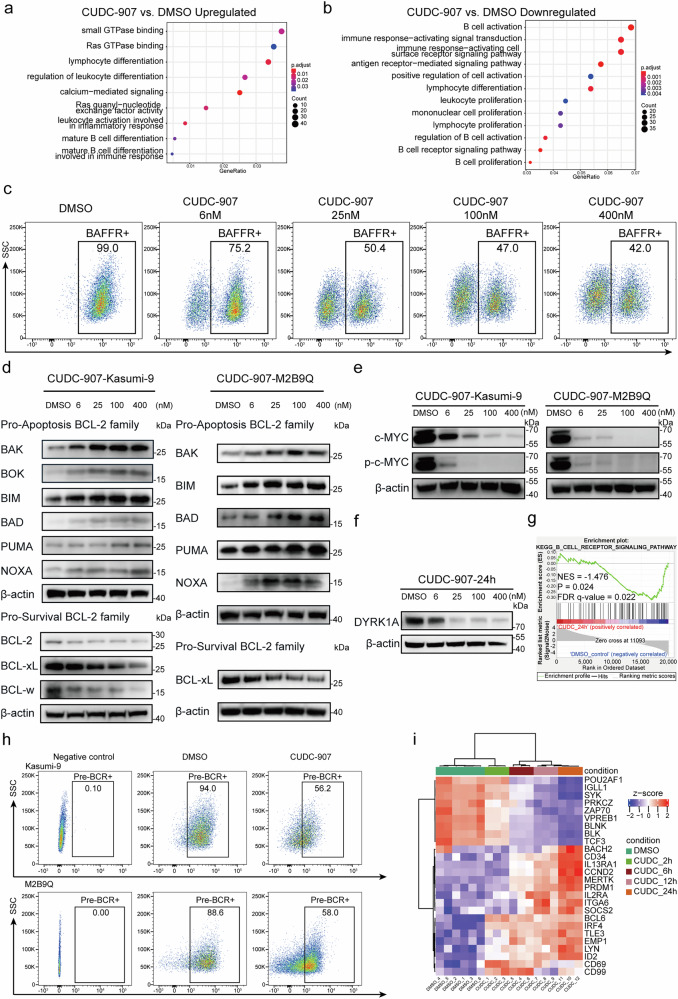
CUDC-907 Disrupted Positive Feedback Loop and Downregulated Expression of MH Fusion Target Genes. **a** GO analysis of up-regulated DEGs in Kasumi-9 cells after 12 h CUDC-907 treatment. **b** GO analysis of down-regulated DEGs in Kasumi-9 cells after 12 h CUDC-907 treatment. **c** Flow cytometry (FCM) analysis of BAFFR expression on the surface of Kasumi-9 cells. **d** CUDC-907 increases protein levels of pro-apoptosis molecules and decreases pro-survival members of the BCL-2 family. **e** CUDC-907 decreases protein expression and phosphorylation levels of c-MYC. **f** Immunoblotting detection and quantification of DYRK1A in Kasumi-9 cells treated with DMSO or CUDC-907 at indicated concentrations for 24 h. **g** GSEA pathways enriched in Kasumi-9 cells treated with CUDC-907 compared to control (DMSO). **h** FCM analysis of pre-BCR expression on the surface of Kasumi-9 and M2B9Q cells treated with CUDC-907 versus control (DMSO). **i** Heatmap depicting gene expression changes after treatment with 25 nM CUDC-907, focusing on components of the pre-BCR signaling pathway aberrantly regulated in pre-BCR + ALL patients

We next performed RNA-seq experiment to profile the comprehensive gene expression changes in Kasumi-9 cells following treatment with CUDC-907 (25 µmol/L) for 2 h, 6 h, 12 h and 24 h, respectively, compared to DMSO control (Supplementary Fig. 4a). The DEGs at each time point were evaluated with stringent criteria (Supplementary Fig. 4b). Taking 12 h of treatment as an example, 2,877 genes were up-regulated and 965 genes were down-regulated (Supplementary Fig. 4c). To explore the potential dysregulated pathways and networks induced by CUDC-907, we performed GO and KEGG enrichment analysis of DEGs. After 12 h of treatment with CUDC-907, the upregulated genes were mainly enriched in small GTPase binding, lymphocyte differentiation, calcium-mediated signaling, mature B cell differentiation and other pathways related to B cell maturation (Fig. 5a). This finding was consistent with acetylation activation by CUDC-907 administration (Fig. 4f). The down-regulated genes were enriched in B cell activation, immune response-activating signal transduction, leukocyte proliferation and B cell receptor signaling pathway (Fig. 5b). The pathway associated with B cell activation was suppressed post-treatment, notably through down-regulation of B cell activating factor receptor (*BAFFR*, *TNFRSF13C*) expression (Fig. 5b). The expression of BAFFR on the surface of Kasumi-9 cells reached to 99% in flow cytometry (FCM) assay (Fig. 5c). The critical targets of BAFF-dependent survival likely result from a favorable balance of upregulated expression of pro-survival BCL-2 family members and downregulated expression of pro-apoptotic BH3-only proteins, thus preventing Bak/Bax-dependent apoptosis.^29,30^ The expression levels of pro-apoptotic proteins BAK, BOK, BIM, BAD, PUMA and NOXA were increased, while pro-survival BCL-2 family members BCL-2, BCL-xL, BCL-w were decreased (Fig. 5d). In addition, the expression and phosphorylation of c-MYC, a key factor closely associated with proliferation and apoptosis in BCP-ALL, were significantly reduced (Fig. 5e). These results suggested that CUDC-907 inhibited the proliferation of leukemia cells in vitro by inducing apoptosis through BAFF-BAFFR-BCL2 family axis (Supplementary Fig. 4d). BCP-ALL cells expressed high levels of BAFFR, which activated the non-canonical NF-kB (ncNF-kB) pathway in response to BAFF in a DYRK1A-dependent manner to maintain the survival of leukemia cells (Supplementary Fig. 4e). Analysis of the Broad Institute’s Cancer Cell Line Encyclopedia (CCLE) revealed that DYRK1A was overexpressed in hematopoietic malignancies, especially in BCP-ALL, relative to other tumor types (Supplementary Fig. 4f, g). BAFFR expression was decreased in Kasumi-9 cells treated with CUDC-907, and a corresponding reduction in DYRK1A expression was also observed (Fig. 5f and Supplementary Fig. 4h). In a previous report, phosphorylation of MEF2D by DRYK1A was found to significantly enhance the protein level of MEF2D while attenuating its transcriptional activity, ultimately leading to reduced transcription of MEF2D target genes.^31^ In this scenario, the question was raised if the inhibition of DYRK1A could reduce the expression levels of both MEF2D and MH. To test this hypothesis, Harmine hydrochloride, an inhibitor of DYRK1A, was used to treat Kasumi-9 cells, showing no significant changes in MEF2D and MH expression (Supplementary Fig. 4i). These findings suggested that DYRK1A was involved in elaborately disrupted regulatory networks in *MEF2D* fusion BCP-ALL, whereas inhibition of DYRK1A alone was insufficient to decrease *MH* expression.

Gene Set Enrichment Analysis (GSEA) analysis revealed a significant down-regulation of the (pre-)BCR signaling pathway and its downstream events following CUDC-907 treatment (Fig. 5g and Supplementary Fig. 4j). FCM detected a significant reduction in pre-BCR proportion after CUDC-907 treatment (Fig. 5h). In pre-BCR + ALL, genes with abnormally high expression such as *VPREB1*, *IGLL1*, *SYK*, *PRKCZ*, *BLNK*, *BLK*, *ZAP70*, *BACH2*, *TCF3* and *POU2AF1* were down-regulated after treatment; while genes that were repressed due to aberrant pre-BCR activation, such as *IL2RA(CD25)*, *IL13RA1*, *CD34*, *ID2*, *CD69*, *CD99*, *ITGA6*, *CCND2*, *SOCS2*, *PRDM1*, *TLE3*, and *EMP1* genes were up-regulated by CUDC-907 (Fig. 5i). Due to the presence of *MEF2D* fusion, B lineage cell differentiation was blocked at pre-B stage. The overexpression of pre-BCR signaling pathway, which is targeted by *MEF2D* fusion, leads to persistent activation of PI3K signaling pathway, resulting in an aberrant positive feedback loop.^32^ CUDC-907 directly inhibited PI3K, thereby disrupting the abnormal regulatory network (Supplementary Fig. 4k). Among the target genes activated by MH fusion, 1348 genes were down-regulated by more than 1.5-fold log2Foldchange after 24-h (hrs) of CUDC-907 treatment, corroborating the results of *MH* fusion gene knockdown in Kasumi-7 cells^10^ (Supplementary Fig. 4l).

### Suppression of leukemia cell growth in vivo and prolongation of the lifespan of *MEF2D* fusion-driven leukemic mice upon effect of CUDC-907

**Fig. 6:**
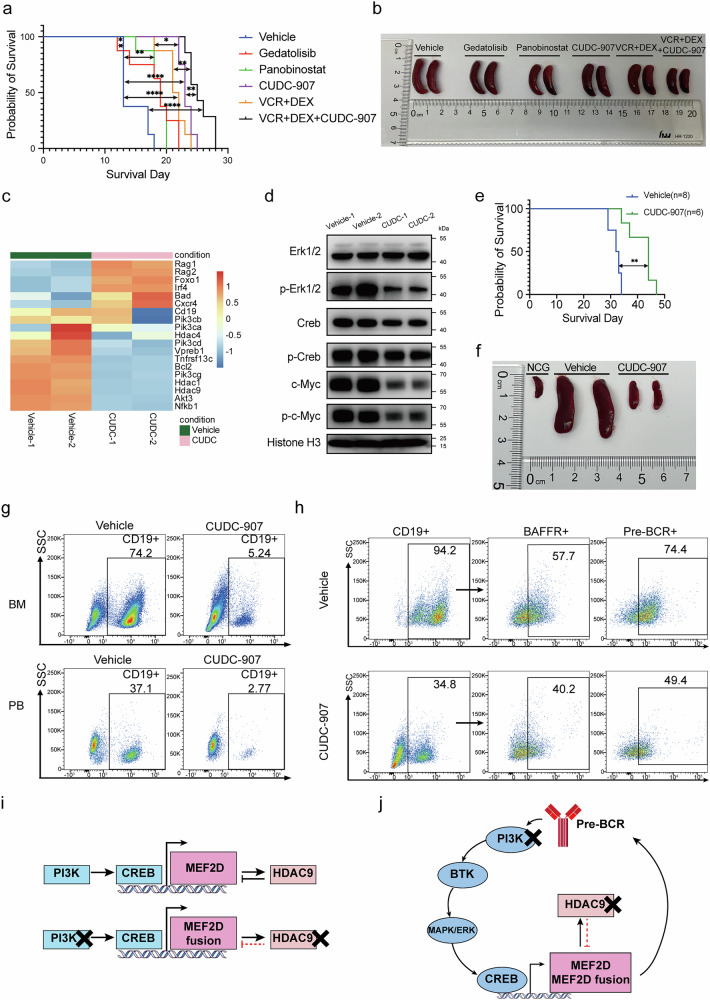
CUDC-907 Significantly Improved Overall Survival of MEF2D Fusion BCP-ALL Mice. **a** Kaplan-Meier survival curves of MH/NRAS^G12D^ recipient mice treated with continuous administration of solvent (control, *N* = 8), Gedatolisib (10 mg/kg, *N* = 8), LBH-589 (2.5 mg/kg, *N* = 8), CUDC-907 (100 mg/kg, *N* = 8), VCR (0.15 mg/kg) plus DEX (1 mg/kg) (*N* = 8), and the combination of these three drugs (*N* = 7) for 2 weeks on a 5-days-on/2-days-off schedule. Error bars represent ± SD. *P < 0.05; **P < 0.01; ***P < 0.001; ****P < 0.0001. **b** Spleen sizes from mice described in panel. **c** Heatmap of expression changes of key genes following CUDC-907 administration in vivo. **d** Western blot analysis of spleen samples from (**a**). **e** Kaplan–Meier survival curves of MB PDX mice treated with vehicle or CUDC-907 (100 mg/kg). **f** Spleen sizes from mice described in (**e**) (*N* = 2). **g** FCM analysis of CD19+ leukemia cells in BM and peripheral blood of MB PDX mice. **h** FCM analysis of BAFFR+ and pre-BCR+ proportions in CD19+ leukemia cells in spleens of MB PDX mice. **i** Diagram illustrating how CUDC-907 (black cross) exerted its dual inhibitory effects on the upstream and downstream signaling molecules of MEF2D fusions. **j** Diagram illustrating how CUDC-907 (black cross) disrupted positive feedback loops in the PI3K-MEF2D fusions-pre-BCR signaling pathway. Note in the lower panel of **i**, the negative feedback effect of HDAC9 on the *MEF2D* gene expression (shown in the upper panel of **i**) during normal B-cell development is deregulated in the case of *MEF2D* fusions (shown as red dot lines)

We utilized the *MH/NRAS*^*G12D*^ BCP-ALL mice to determine efficacy in vivo.^12^ First, we conducted a dose escalation experiment to determine the optimal dosage of CUDC-907 for in vivo treatment. Intragastric administration of CUDC-907 was initiated on the third day after transplantation using different doses, 25 mg/kg/d, 50 mg/kg/d, and 100 mg/kg/d, with solvent as control. Even at the lowest dose, CUDC-907 significantly prolonged mice survival, and increasing the dosage resulted in further extension of median survival time (Supplementary Fig. 5a). Meanwhile, CUDC-907 showed acceptable level of adverse effects such as weight loss (less than 10%) (Supplementary Fig. 5b). To examine the therapeutic effect of CUDC-907 relative to conventional chemotherapy and HDAC or PI3K single target drug, mice were divided into six groups and given distinct treatments: solvent control, 10 mg/kg Gedatolisib, 2.5 mg/kg Panobinostat, 100 mg/kg CUDC-907, 0.15 mg/kg Vincristine (VCR) plus 1 mg/kg Dexamethasone (DEX), and the combination of CUDC-907 with VCR + DEX. Survival analysis showed modest improvements with Gedatolisib and Panobinostat, while VCR + DEX was more effective. Notably, CUDC-907 alone demonstrated more potent survival benefits than VCR + DEX (Fig. 6a). After 2 cycles of administration, tissue samples were harvested and revealed that splenomegaly was alleviated in all treatment groups except for Gedatolisib group (Fig. 6b and Supplementary Fig. 5c). FCM analysis of BM cells showed the similar tendencies of reduction of GFP + B-lymphoblasts under the 4 treatment protocols, with the triad combination therapy achieving the most significant efficacy (Supplementary Fig. 5d). We performed RNA-seq and western blot analyses on spleen cells and observed that the modulation of key molecules following CUDC-907 administration in vivo was consistent with the aforementioned findings in vitro (Fig. 6c, d). The immunohistochemistry (IHC) assay demonstrated a significant reduction of *Cd19*, *Pik3ca*, *Hdac1*, *Hdac9* and *Bcl-2* levels in the CUDC-907-treated group, validating the result we obtained on cell lines (Supplementary Fig. 5e). Hematoxylin and eosin (HE) staining showed spleen structure destruction in the control group, partial preservation in single-target drug and VCR + DEX groups, and near-intact structure in the VCR + DEX + CUDC-907 group (Supplementary Fig. 5f). Overall, treatment with CUDC-907 effectively suppressed leukemia cell proliferation and significantly reduced tumor burden as well as infiltration across various organs.

In *MB* PDX model, CUDC-907 administration significantly prolonged the lifespan of *MB* fusion BCP-ALL mice from 32.5 days to 44 days (Fig. 6e). We sampled the mice at the same time point 3 weeks after CUDC-907 treatment, and found that the mice in the solvent group had obvious splenomegaly and hepatomegaly due to extensive infiltration of leukemia cells. Mice treated with CUDC-907 did not display noticeable swelling in spleens or livers as compared to healthy mice (Fig. 6f, Supplementary Fig. 5g). In HE staining of pathologic sections, the solvent group showed pronounced leukemia cell infiltration in the spleen and liver, disrupting organ’s architectural integrity. By contrast, CUDC-907 treatment resulted in minimal leukemia cell presence while most organic structures were preserved (Supplementary Fig. 5h). FCM analysis revealed predominance of CD19+ leukemia cells in BM, accounting for 74.2% in vehicle-treated mice, compared to only 5.24% in those treated with CUDC-907. Peripheral blood analysis showed that CD19+ leukemia cell percentage was 37.1% for vehicle-treated mice and decreased to 2.77% when treated with CUDC-907 (Fig. 6g). In spleen cells, CD19+ leukemia cell proportion was 94.2% for vehicle-treated group and reduced to 34.8% for CUDC-907-treated group. Treatment with CUDC-907 resulted in a decrease in BAFFR and pre-BCR expression in splenic CD19+ leukemia cells, providing further support for our previous mechanistic study results at the cellular level (Fig. 6h). In addition, we conducted in vivo experiments using AKT and BCL2 inhibitors, namely Triciribine and Venetoclax. Although both of them exhibited good efficacy in vitro, neither of them showed comparable therapeutic effects to CUDC-907 in vivo, highlighting the significance of the dual-target strategy (Supplementary Fig. 6a–d). These findings provided critical evidence that CUDC-907 is capable of alleviating tumor burden in vivo and represents a novel therapeutic option for *MEF2D* fusion (+) BCP-ALL.

## Discussion

*MEF2D* fusion (+) BCP-ALL is an ALL subtype characterized by the abnormal upregulation of *HDAC9* and expression of pre-BCR on cell surface.^1,2,7,10,12^ Due to the poor prognosis, it is crucial to identify targeted therapeutic strategies for this patient group.^8^ Previous treatment studies, which primarily focused on HDAC9 as a key phenotypic feature, have explored the use of HDAC inhibitors.^7,8,12,15^ MEF2D regulates the transcriptional activity of *HDAC9*, and when fused with partner, the negative feedback mechanism controlling *HDAC9* expression is disrupted, leading to its overexpression (Fig. 6i-j).^9,13^ Fusion partners at the C-terminus, such as *HNRNPUL1*, further exacerbate this dysregulation.^12^ Direct inhibition of HDAC expression specifically targets the primary focus of *MEF2D* fusion. However, HDAC inhibitors must be combined with conventional chemotherapy agents to demonstrate efficacy in extending survival in leukemic mice.^12^ This observation underscores the limitations of targeting HDAC alone and suggests the involvement of additional pathogenic factors and pathways in the pathogenesis of *MEF2D* fusion BCP-ALL.

Indeed, the BCP-ALL with distinct *MEF2D* fusions also relies on hyperactivation of the pre-BCR signaling pathway to evade apoptosis and maintain the leukemogenic ability.^10^ Previously, our group identified a signature gene set related to *MH* (+) patients, which could be used for disease stratification even beyond *MEF2D* fusion BCP-ALL.^33^ In both *MH* and *MB* fusion cell systems, histone H3 acetylation activated the PI3K/AKT signaling pathway while multiple genes involved in this cascade were located in SEs (Fig. 3a–d). Suppression of *MH* fusion gene expression led to downregulation of PI3K/AKT pathway activity and triggered leukemia cell death (Fig. 3f). Collectively, these results indicated an intimate interdependence between *MEF2D* fusion and the PI3K/AKT signaling pathway, with activated pathways playing a critical role in *MEF2D* fusion BCP-ALL pathogenesis. Notably, drug screening results identified the PI3K signaling pathway acted as a pivotal switch governing the fate of leukemia cells downstream of pre-BCR activation. By employing a large-scale drug screening, we discovered that inhibitors of PI3K pathway, MAPK pathway, BCL2 family, and HDAC were found to significantly suppressed in vitro proliferation of leukemia cells harboring the *MEF2D* fusion gene (Fig. 1d and Supplementary Fig. 1b, c). These data confirmed previous mechanistic studies, validated the feasibility of precision therapies through drug screening, and provided new insights into the regulatory network in *MEF2D* fusion BCP-ALL pathogenesis.

Among the hits identified in the drug screening, we observed that CUDC-907, a dual-target inhibitor of PI3K and HDAC, effectively suppressed leukemia cell proliferation by more than 90% in both *MH* and *MB* cells at nanomolar concentrations (Fig. 2b and Supplementary Fig. 1a). CUDC-907 exhibited inhibitory effects on HDAC activity and downregulated the expression of various HDAC member genes (Supplementary Fig. 3c). Moreover, the compound exerted its pharmacological impact by targeting the pre-BCR-PI3K signaling pathway that was crucial in *MEF2D* fusion leukemia cell survival (Figs. 4a, b and 6i). Inhibition of this pathway led to reduced transcriptional activity of the PI3K-CREB axis responsible for regulating WT *MEF2D* and *MEF2D* fusion gene expression (Figs. 4a, b and 6j). Consequently, numerous target genes associated with *MEF2D* fusion, including those within the pre-BCR pathway that form a positive feedback loop, were downregulated upon effect of CUDC-907 (Fig. 6j). Remarkably, the pharmacological effect of CUDC-907 was comparable to that of directly knocking out the pathogenic *MEF2D* fusion in leukemia cell lines^10^ (Supplementary Fig. 4j). By simultaneously inhibiting both upstream regulatory factors and downstream target genes, CUDC-907 disrupted aberrant regulatory loop effectively and eliminated key pathogenic factors associated with *MEF2D* fusions.

CUDC-907 triggered apoptosis in *MEF2D* fusion (+) leukemia cells by upregulating pathways related to differentiation and apoptosis (Figs. 4f and 5a). During the downregulation of B cell activation pathway, BAFF, a member of the tumor necrosis factor superfamily (TNFSF), facilitates B cell proliferation and survival.^34^ It is also well known to interact with three distinct receptors on B cells, namely BAFFR, BCMA (B-cell maturation antigen), and TACI (transmembrane activator, calcium-modulator, and cyclophilin ligand interactor).^29,35,36^ BAFFR is expressed on immature and mature B cells, but is not expressed on normal pre-B cells.^35,37^ However, pre-BCR + ALL lymphocytes express simultaneously BAFF and BAFFR, which, when ligated, can activate both canonical and non-canonical pathways of NF-κB signaling to promote tumor cell survival in vitro.^35,38,39^ CUDC-907 suppressed BAFFR expression and modulated downstream BCL2 family members to induce apoptosis in leukemia cells (Fig. 5c, d).^30^ DYRK1A, a mediator through BAFF-induced ncNF-kB activation to promote autoimmunity and B-cell leukemogenesis, was downregulated after CUDC-907 administration (Fig. 5e).^40,41^ A subtype of BCP-ALL characterized by amplification of chromosome 21 and a common region exhibiting gene amplification and overexpression in ALL has been identified.^42^ The genes related to the pathogenesis of leukemia within this region involves *DYRK1A*. *DYRK1A* is a kinase essential for normal lymphopoiesis that mediates the degradation of cyclin D3 after phosphorylation, thereby facilitating lymphocyte cell-cycle exit and subsequent lymphoid maturation.^43^ The PI3K pathway has recently been demonstrated to implicate in BAFFR function.^29,38,44^ Several components of the PI3K signaling network can activate downstream effectors of BAFFR, while PI3K activation is capable of promoting c-REL-dependent upregulation of BAFFR, creating an additional positive feedback loop.^29^ Thus, the positive feedback loop could be disrupted by inhibiting the PI3K target.

Dual targeting of core pathways by CUDC-907 significantly enhanced in vivo efficacy. Monotherapy with CUDC-907 not only outperformed single-target inhibitors of HDAC and PI3K but also significantly extended the survival of leukemia-bearing mice compared to the traditional chemotherapy combination of vincristine and dexamethasone (Fig. 6a). When combined with chemotherapy drugs, the therapeutic effect of CUDC-907 was further enhanced, demonstrating superior tumor burden reduction (Fig. 6b). CUDC-907 has exhibited antitumor activity in various hematological malignancies, including diffuse large B-cell lymphoma, AML, CLL, and multiple myeloma.^45–48^ In this work, we demonstrated a strong anti-proliferative effect of this compound on primary BM cells from BCP-ALL patients or PDX models harboring *MEF2D* fusion genes. Its therapeutic potential for *MEF2D* fusion (+) BCP-ALL was confirmed by in vivo assays. Since a proportion of pre-BCR + BCP-ALL cases without *MEF2D* fusion but disposing related signature gene set,^33^ it is reasonable to speculate that CUDC-907 may exhibit comparable efficacy in pre-BCR + BCP-ALL patients, given the analogous transcriptomic expression profiles and shared pathogenic mechanisms.

## Materials and methods

### Cell line and ex vivo cell model

Kasumi-9 cell line derived from a BCP-ALL patient with *MH* was purchased from Japanese Collection of Research Bioresources cell bank. Cells were cultured in RPMI 1640 medium supplemented with 20% fetal bovine serum (FBS) in the presence of penicillin/streptomycin.

M2B9Q ex vivo cell model was derived from the PDX model with *MEF2D::BCL9* fusion. We engrafted the murine spleen cells into NCG mice to establish a second-generation PDX model and collect and culture leukemia cells ex vivo. The culture medium consisted of 20% FBS, RPMI 1640, and GlutaMax in the presence of penicillin/streptomycin. The specific cell model construction method referred to previously published procedures.^15^

PDX model related animal experiments conducted in this study were formally approved by the Institutional Animal Care and Use Committee (IACUC) of Crown Bioscience, in accordance with the ethical guidelines for animal experimentation.

Cells were maintained in a humidified incubator at 37 °C with 5% CO_2_.

### High-throughput drug screening

The Kasumi-9 and M2B9Q cells were seeded in 50 µL culture media in 384-well (4 × 10^3^ cells/well) plates. 0.1 µL of either a 200 or 40 mmol/L drug library was added to the cells using an Automated Liquid Handling Platform. The cells were then incubated for 5 days before adding CellTiter-Glo reagent with the Multidrop Combi. Luminescence readings were recorded using an SpectraMax L chemiluminescence microplate reader. Cell viability was calculated as a percentage relative to DMSO-treated cells.

### Bone marrow samples from patients

BM samples from BCP-ALL patients were obtained under informed consent from Ruijin Hospital and Shanghai Children’s Medical Center (SCMC) affiliated to Shanghai Jiao Tong University School of Medicine. Mononuclear cells were enriched by density gradient centrifugation with Ficoll solution. The use of samples from Ruijin Hospital were approved by the Ethics Committee of Ruijin Hospital under NO.KY2022-117. Patients from SCMC were in the cohort of Chinese Children’s Cancer Group (CCCG)-ALL-2015 trial (clinical trial number: ChiCTR-IPR-14005706) and CCCG-ALL-2020 (clinical trial number: ChiCTR2000035264). The studies were approved by the Institutional Review Board of SCMC, (NO.SCMCIRB-K2024169-1, SCMCIRB-K2020076-5). All relevant ethical regulations were followed in this study.

### ChIP-seq and data analysis

Kasumi-9 and M2B9Q cells with or without 100 nM CUDC-907 treatment for 24 h were collected. ChIP DNA was prepared by using the VAHTS DNA Clean Beads (Vazyme). Immunoprecipitation was performed with the antibodies against H3K9Ac and H3K56Ac (abclonal). The DNA libraries were constructed by using the VAHTS Universal Pro DNA Library Prep Kit (Vazyme) and sequenced on the MGISEQ-2000 (BGI).

Reads were aligned to the human genome build hg19 using Bowtie 2 (version 2.5.1). PCR duplicates were marked using the MarkDuplicates function in Picard-tools. Filtration and removing background reads using samtools (1.16.1). MACS (2.2.9.1) was used to identify H3K9ac peaks of M2B9Q and H3K27ac peaks of Kasumi-7 with a *P* value of 10^−5^ and 10^−9^,^10^, respectively. Differential peaks with a cutoff of 3. Creating normalized bigWig alignments using deeptools (3.3.0). The Integrative Genomics Viewer (IGV version 2.9.4) was used to visualize the data. Gene annotation and distributions of ChIP-seq peaks overlapping specific gene features were analyzed using the R package ChIPseeker (v1.38.0). SE regions were identified by Rank Ordering of Super Enhancers program to ChiP-seq reads. A stitching distance of 12.5 kb, promoter exclusion zone encompassing ±2 kb around the TSSs, and default parameters were used. The SEs were assigned to the gene for which the TSS was nearest to the center of the stitched enhancer.

### In vivo experiments

3 × 10^5^ spleen cells from *MH/NRAS*^*G12D*^ BCP-ALL mice of secondary transplantations were transplanted into sublethal irradiated (2 Gy) recipient mice.^12^ The *MH/NRAS*^*G12D*^ tertiary recipient mice were randomly divided into 6 different treatment groups, including a control group receiving vehicle only, intraperitoneal administration of 10 mg/kg PI3Ki Gedatolisib,^49^ intraperitoneal administration of 2.5 mg/kg HDACi LBH-589,^12^ oral gavage administration of 100 mg/kg HDAC/PI3Ki CUDC-907, intraperitoneal administration of 0.15 mg/kg VCR once a week and 1 mg/kg DEX, and combination therapy with CUDC-907 plus VCR + DEX for 4 cycles 5-days-on/2-days-off starting from day 3 after transplantation. Gedatolisib (CSNpharm, CSN16214) was diluted in 6.25% DMSO, 40% PEG 300, 2% Tween 80, and double-distilled water (ddH_2_O). LBH-589 (Selleck, S1030) was diluted in 2% DMSO, 48% PEG 300, 2% Tween 80, and ddH_2_O. CUDC-907 (CSNpharm, CSN16258) was reconstituted in 30% captisol.^50^ VCR (Selleck, S1241) was diluted in ddH_2_O. DEX (Selleck, S1322) was diluted in 5% DMSO, 45% PEG 300, and ddH_2_O. Mice were weighed daily before drug administration. The *MH/NRAS*^*G12D*^ BCP-ALL mice experiments were formally approved by Ethics Committee and Animal Care Committee of Ruijin Hospital.

### Statistical analyses

All data were analyzed using GraphPad Prism 9.0. The results were expressed as the mean $\pm \,$SD. Significant changes between 2 groups were analyzed with an unpaired, two-tailed Student t test. One-way ANOVA was applied to judge significant differences among multiple groups. Kaplan-Meier analyses were performed, and the log-rank Mantel-Cox test was used to determine statistical difference between the survival curves of two groups. *P* < 0.05 was considered statistically significant. The level of significance was indicated as *P < 0.05; **P < 0.01; ***P < 0.001; ****P < 0.0001.

Detailed information of primers and antibodies used and supplementary methods can be found in the Supplementary files.

## Supplementary information

Revised Supplementary File

Supplementary Table

Gating strategies

Uncropped blots-1

Uncropped blots-2

Uncropped blots-3

## Data Availability

The ChIP-seq, ATAC-seq, and RNA-seq data of Kasumi-7 have been deposited in ArrayExpress under the following accession numbers: E-MTAB-8466, E-MTAB-8463, E-MTAB-8491, and E-MTAB-8480. The RNA-seq and acetylated ChIP-seq data before and after CUDC-907 treatment of Kasumi-9 and M2B9Q have been submitted in BioProject database under BioProject ID PRJNA1243691. RNA-seq analyses following CUDC-907 administration in *MH/NRAS*^*G12D*^ mice have been submitted under BioProject ID PRJNA1243720.
