# Supplementary material for: Molecular mechanisms of unique therapeutic potential of CUDC-907 for MEF2D fusion-driven BCP-ALL
Source: Signal Transduct Target Ther. 2025 Jul 23;10:230. doi: 10.1038/s41392-025-02310-y (PMC12283968; doi:10.1038/s41392-025-02310-y)
Supplement: Supplementary file 1 — Revised Supplementary File [file 41392_2025_2310_MOESM1_ESM.docx]

**Supplementary Materials for**

Molecular Mechanisms of Unique Therapeutic Potential of CUDC-907 for *MEF2D* Fusion-driven BCP-ALL

**Authors:** Qing Xue^1,#^, Ming Zhang^1,#,*^, Yixiao Mo^2,#^, Bo Jiao^1,#^, Xuan Liu^1,#^, Minghao Jiang^1^, Yu Zhou^1^, Yun Tan^1^, HuiMin Li^1^, Jianming Zhang^1^, Qianqian Zhang^1^, Yunqi Li^1^, Jianfeng Li^1^, Xiaofang Ma^1^, Duo-Hui Jing^1^, Jian-Qing Mi^1^, Jin Wang^1^, Zhu Chen^1^, Shu-Hong Shen^2,*^, Sai-Juan Chen^1,*^

Correspondence to Shu-Hong Shen ([shenshuhong@scmc.com.cn](mailto:shenshuhong@scmc.com.cn)), Ming Zhang ([memoryzm91@163.com](mailto:memoryzm91@163.com)) and Sai-Juan Chen ([sjchen@stn.sh.cn](mailto:sjchen@stn.sh.cn))

**This PDF file includes:**

Supplementary Figure 1-6 with their legends

Supplementary Methods


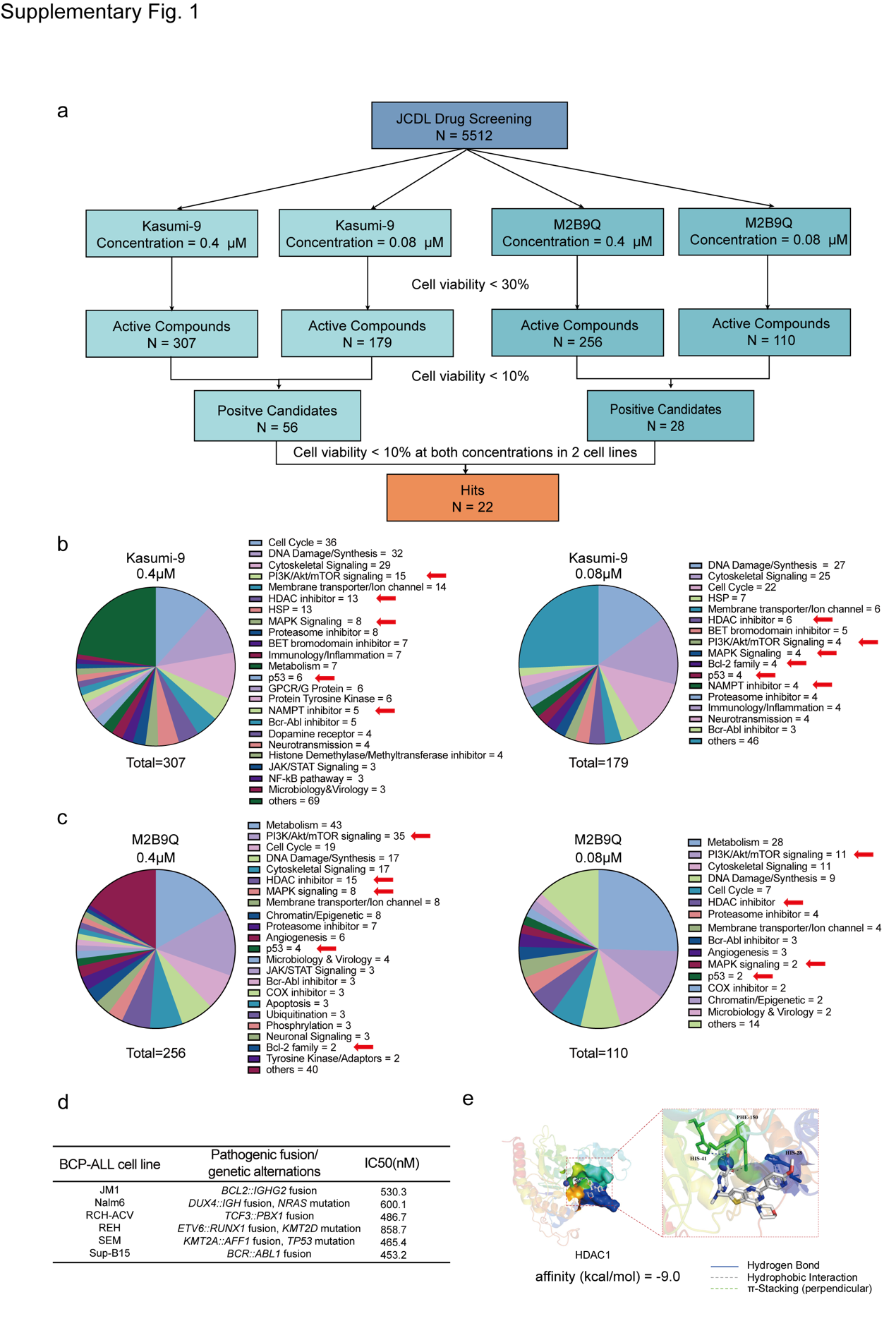


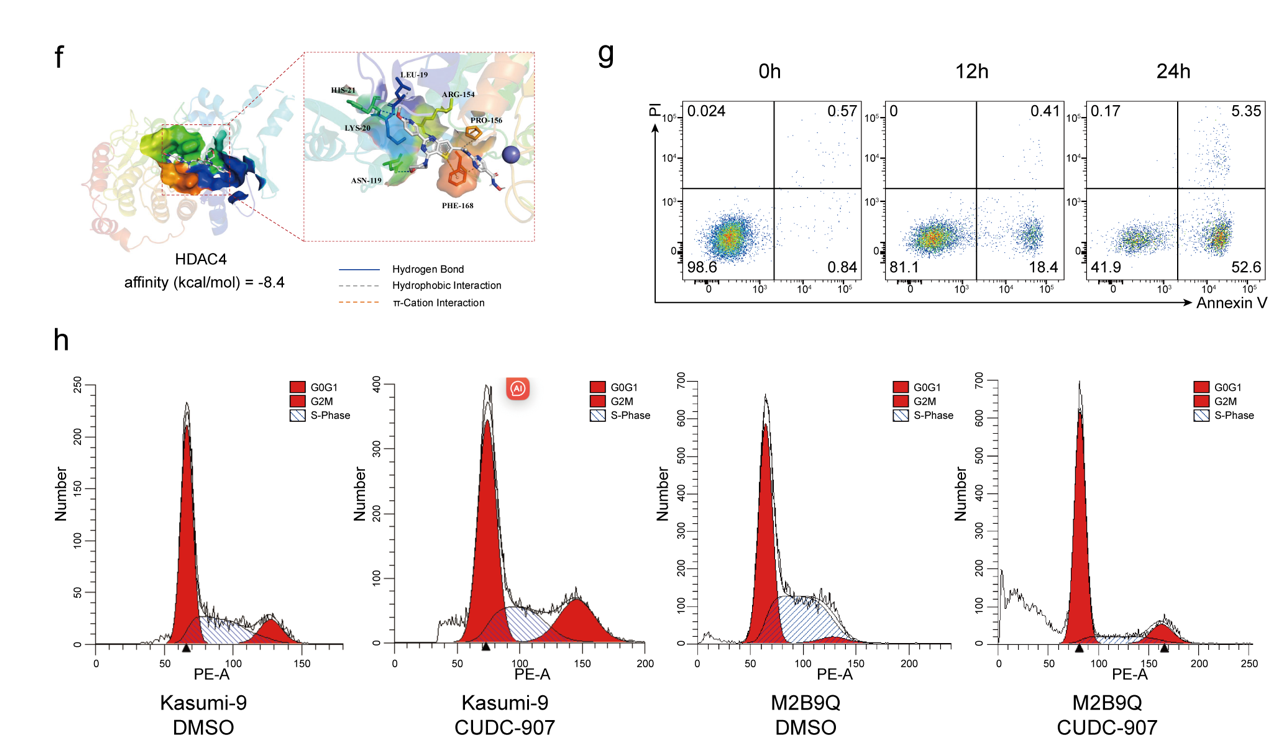


**Supplementary Figure 1. High-Throughput Drug Screening Using MEF2D Fusion Cells.**

**a** Overview of drug screening pipeline. A total of 5,512 compounds were screened, identifying 172 compounds with less than 30% viability across *MH* and *MB* cells at both concentrations, termed 'active compounds'. Drugs with intolerable side effects or lacking dose-response were excluded. The top 22 hits underwent secondary validation using an 8-point dose-response assay in duplicates.

**b** Pie charts showing targets of active compounds that inhibited cell viability by more than 70% at concentrations of 0.4μM and 0.08μM in Kasumi-9 cells. Targets appearing less than three times were categorized into the 'others' group.

**c** Pie charts showing targets of active compounds that inhibited cell viability by more than 70% at concentrations of 0.4μM and 0.08μM in M2B9Q cells. Targets appearing less than three times were categorized into the 'others' group.

**d** IC50 values and pathogenic fusion genes of BCP-ALL cell lines.

**e** The three-dimensional and two-dimensional interaction profiles between the ligand (CUDC-907) and HDAC1 protein through molecular docking.

**f** The three-dimensional and two-dimensional interaction profiles between the ligand (CUDC-907) and HDAC4 protein through molecular docking.

**g** Analysis of apoptosis assessed by annexin-V/PI staining in Kasumi-9 cells treated with CUDC-907 for 12 hours or 24 hours, with corresponding DMSO controls (N=3).

**h** Cell cycle analysis determined by propidium iodide (PI) staining in Kasumi-9 and M2B9Q cells treated with CUDC-907 (100 nM) or DMSO control for 24 hours (N=3).


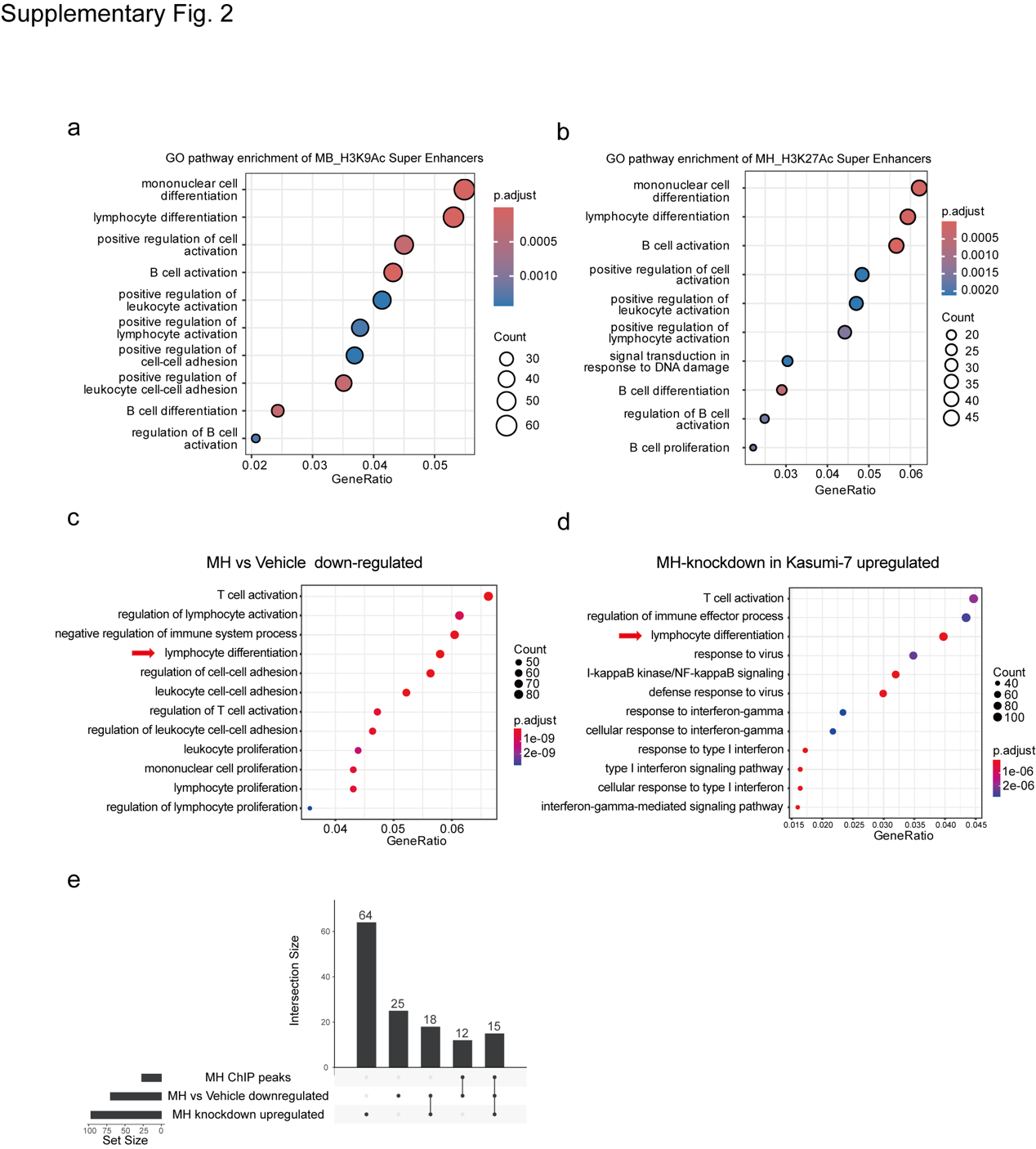


**Supplementary Figure 2. Activation of Multiple Pathways related to Leukocyte Apoptosis by CUDC-907 Treatment.**

**a** GO pathway analysis of genes assigned to SEs involving MEF2D::BCL9 occupancy.

**b** GO pathway analysis of genes assigned to SEs involving MEF2D::HNRNPUL1 occupancy.

**c** The GO pathway analysis diagram of the differentially expressed genes (DEGs) down-regulated in REH cells expressing *MH* fusion compared with the vehicle.

**d** GO pathway analysis of DEGs upregulated after knockdown of the *MH* fusion gene in Kasumi-7 cells.

**e** The intersection of genes downregulated upon expression of the *MH* fusion gene in REH cells, genes upregulated following knockdown of the *MH* fusion gene in Kasumi-7 cells, and MH ChIP binding peaks associated with lymphocyte differentiation.


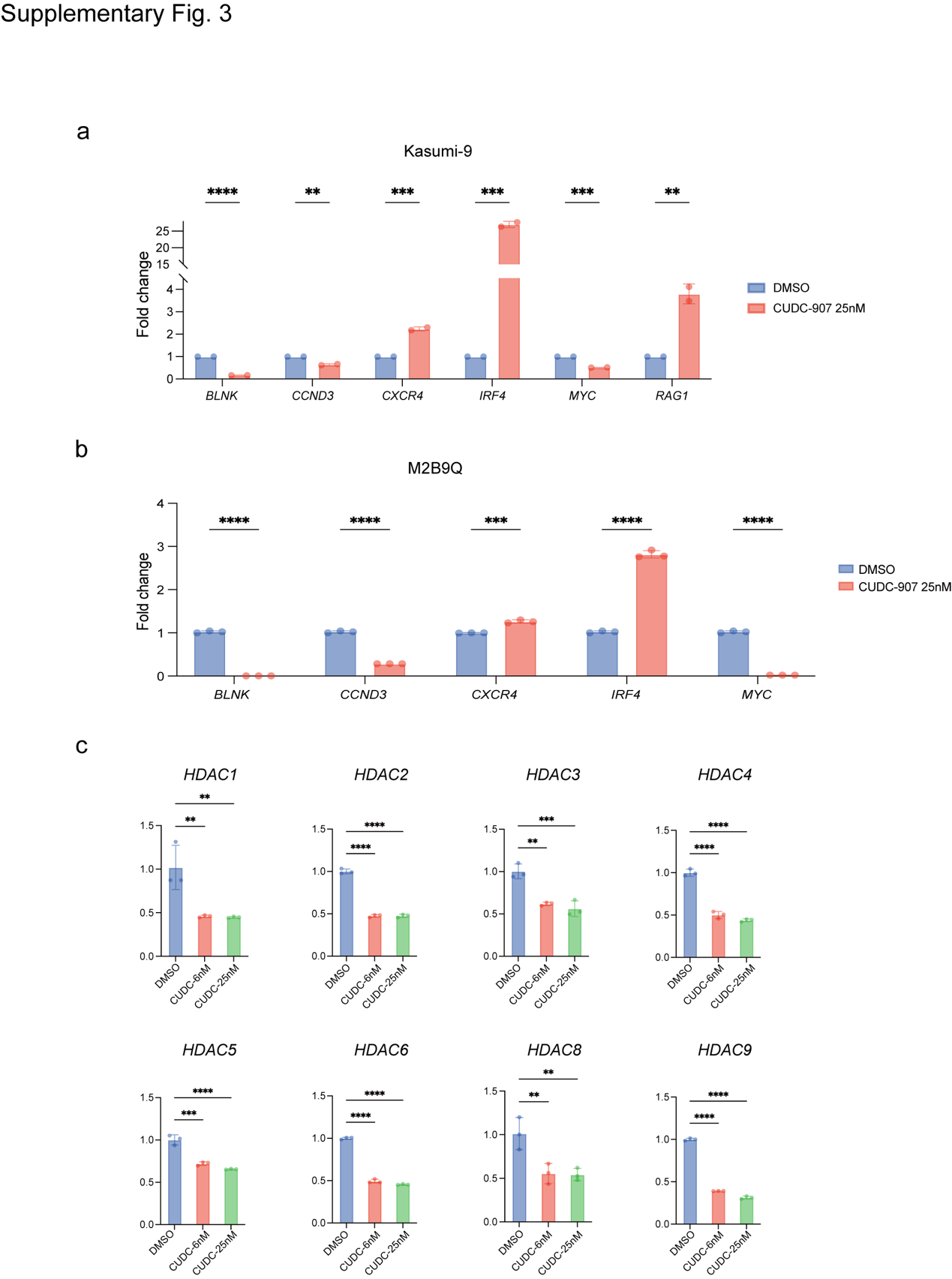


**Supplementary Figure 3. CUDC-907 Regulated PI3K Downstream Target Genes and HDAC Family Genes.**

**a** mRNA expression of FOXO1 target genes *BLNK*, *CCND3*, *CXCR4*, *IRF4*, *MYC* and *RAG1* measured by qRT-PCR in Kasumi-9 cells treated with DMSO or 25nM CUDC-907 for 24 hours, respectively (N=3).

**b** mRNA expression of FOXO1 target genes *BLNK*, *CCND3*, *CXCR4*, *IRF4* and *MYC* measured by qRT-PCR in M2B9Q cells treated with DMSO or 25nM CUDC-907 for 24 hours, respectively (N=3).

**c** mRNA expression of each isoform of *HDACs* measured by qRT-PCR in Kasumi-9 cells treated with DMSO, 6nM, or 25nM CUDC-907 (N=3). Error bars represent ± SD. *P < 0.05; **P < 0.01; ***P < 0.001; ****P < 0.0001.


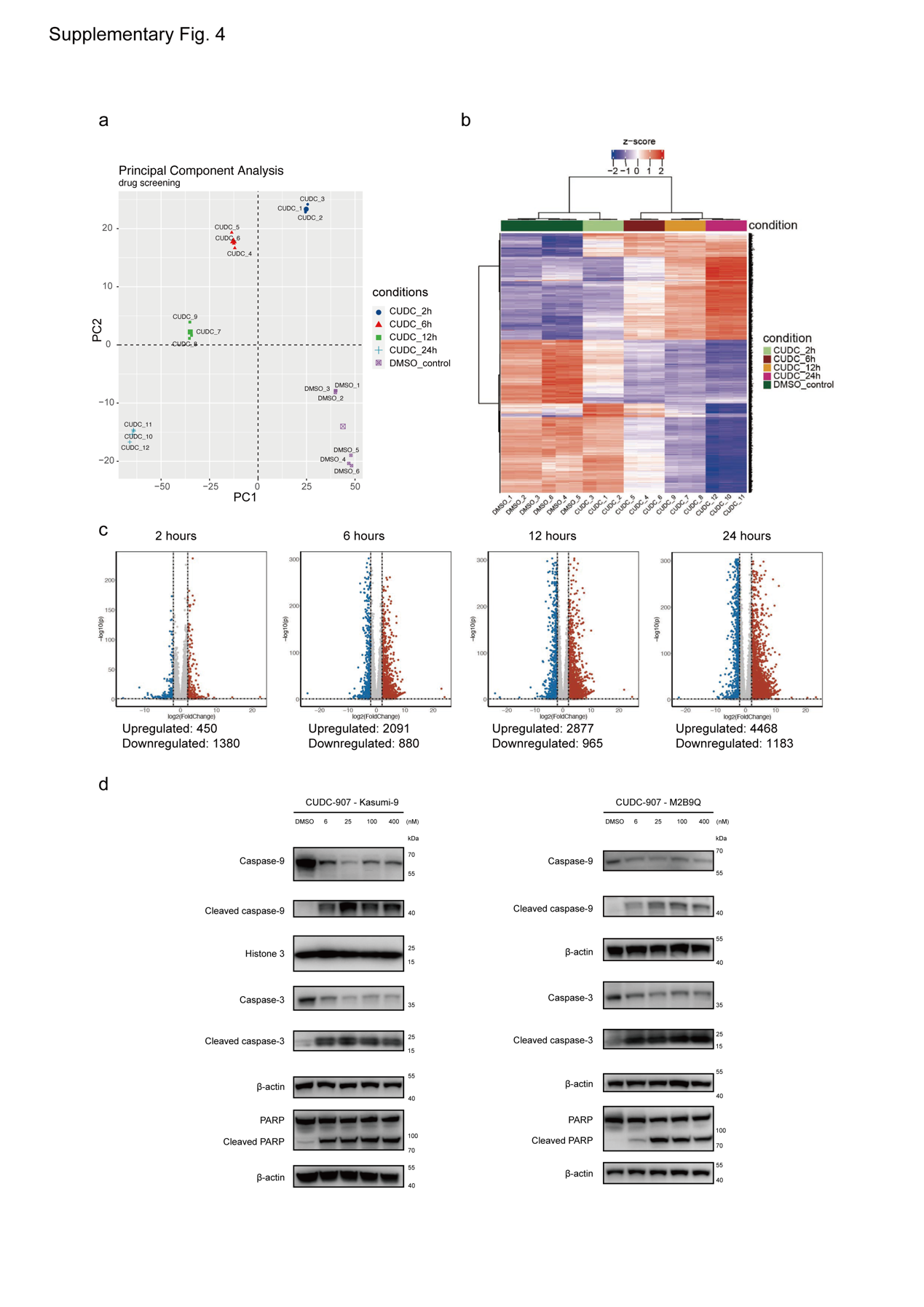


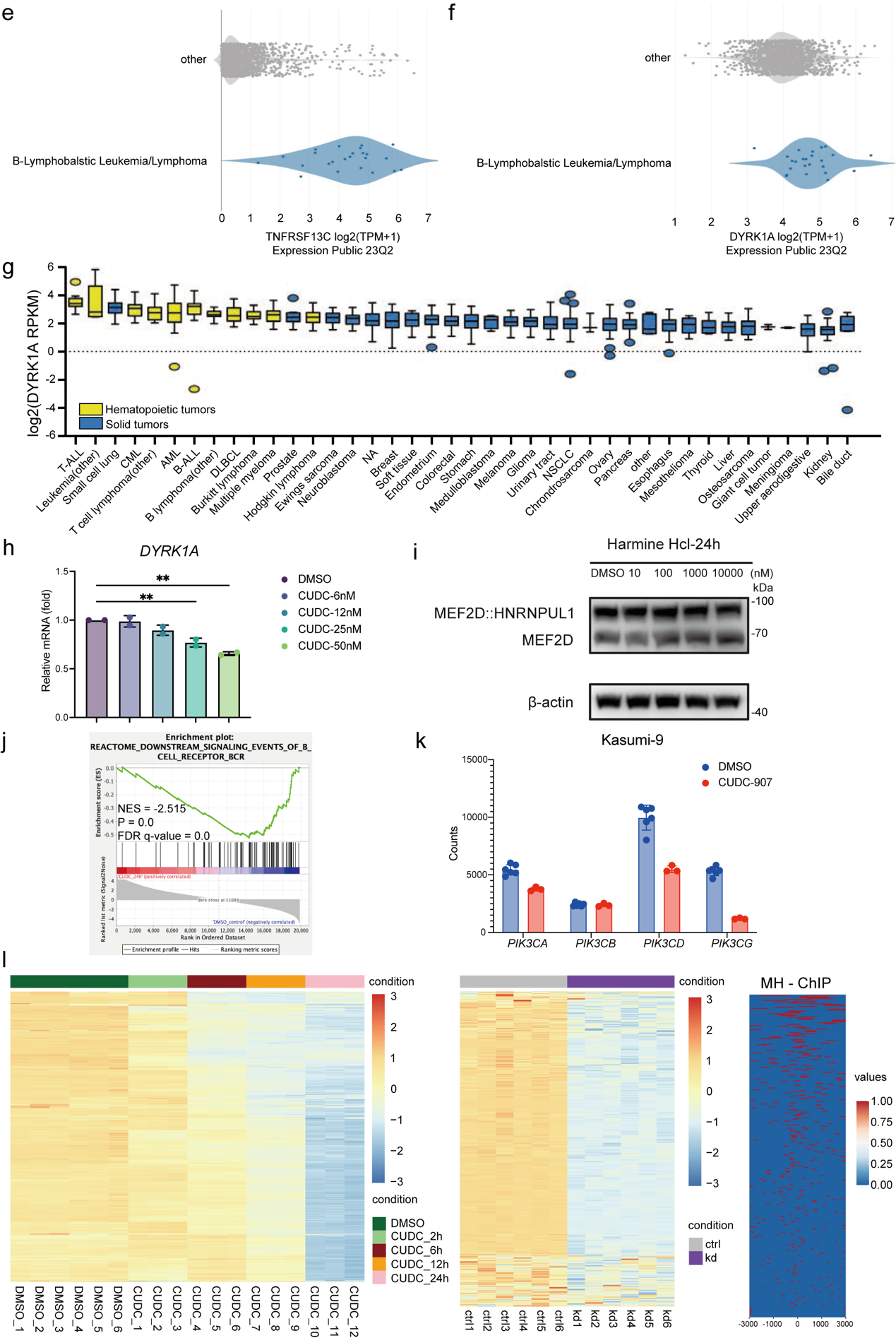


**Supplementary Figure 4. CUDC-907 Regulation of Transcriptional Activity in Kasumi-9 Cells.**

**a** Principal component analysis of Kasumi-9 cells treated with CUDC-907 at different time points, with DMSO as control.

**b** Unsupervised clustering analysis of Kasumi-9 cells treated with CUDC-907 at different time points, with DMSO as control.

**c** Volcano plot showing up- and downregulated genes in Kasumi-9 cells treated with CUDC-907 at different time points compared to DMSO control (fold change > 2.0, p < 0.05).

**d** Validation of activation of mitochondrial apoptosis by western blot analysis for caspase-9, caspase-3 and PARP cleavage in Kaumi-9 and M2B9Q leukemic cells treated with CUDC-907.

**e** Distribution of *TNFRSF13C* mRNA expression (Transcripts Per Million) across cell lines from the Expression Public 23Q2 database, and the comparison of its expression in B-Lymphoblastic Leukemia/Lymphoma to other cancer types.

**f** Distribution of *DYRK1A* mRNA expression (Transcripts Per Million) across cell lines from the Expression Public 23Q2 database, and the comparison of its expression in B-Lymphoblastic Leukemia/Lymphoma to other cancer types.

**g** Distribution of *DYRK1A* mRNA expression (reads per kilobase per million mapped reads [RPKM]) across cell lines from the Broad Institute’s CCLE, ordered by median *DYRK1A* expression levels (dotted lines), interquartile range (IQR) (box), and up to 1.5 times the IQR (bars). NA, not assigned lineage.

**h** *DYRK1A* mRNA expression changes in Kasumi-9 cells after treatment with CUDC-907 at indicated dosages or time points (N=3). Error bars represent ± SD. *P < 0.05; **P < 0.01; ***P < 0.001; ****P < 0.0001.

**i** Immunoblotting detection and quantification of MEF2D and MEF2D::HNRNPUL1 fusion in Kasumi-9 cells treated with DYRK1A inhibitor Harmine hydrochloride for 24 hours.

**j** Enrichment plots of selected Gene Set Enrichment Analysis (GSEA) pathways enriched in Kasumi-9 cells treated with CUDC-907 compared to control (DMSO).

**k** CUDC-907 downregulates the expression of all PI3K isoforms in Kasumi-9 cells.

**l** Downregulation of MH target genes (right) after CUDC-907 treatment (left), consistent with *MH* fusion knock-down (middle) in Kasumi-7 cells.


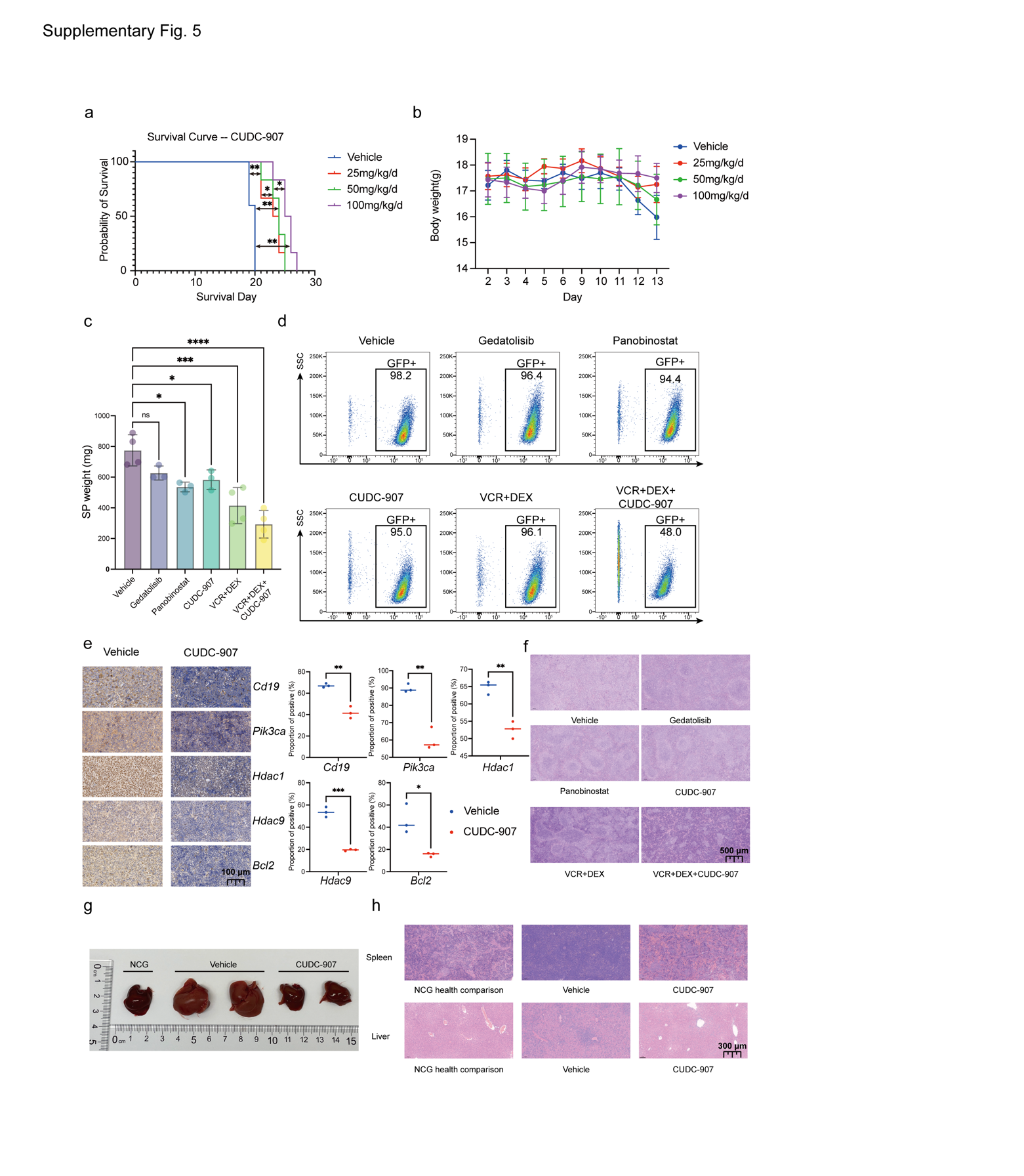


**Supplementary Figure 5.** In Vivo Efficacy of CUDC-907 in both of MH and MB Mouse Models**.**

**a** Kaplan-Meier survival analysis of *MH/NRAS^G12D^* mice treated with vehicle or CUDC-907 at indicated dosages (N=8).

**b** Body weight of mice treated with vehicle or CUDC-907 at indicated dosages (N=6).

**c** Spleen weight of mice in various treatment groups (N=3).

**d** Percentage of GFP-positive cells in bone marrow from *MH/NRAS^G12D^* recipient mice treated with solvent (control), Gedatolisib (10 mg/kg), LBH-589 (2.5 mg/kg), CUDC-907 (100 mg/kg), VCR (0.15 mg/kg) plus DEX (1 mg/kg), and the combination of the three drugs, respectively, for 2 weeks on a 5-days-on/2-days-off schedule.

**e** Immunohistochemical results of Figure 6**c** and quantification using IHC Profiler (N=3). Scale bar: 100μm.

**f** Hematoxylin and eosin-stained sections of spleens from panel 5**b**. Scale bar: 500μm.

**g** Liver sizes from mice described in panel 4**d**.

**h** Hematoxylin and eosin-stained sections of MB PDX spleens and livers treated with CUDC-907 or vehicle. Scale bar: 300μm.

**
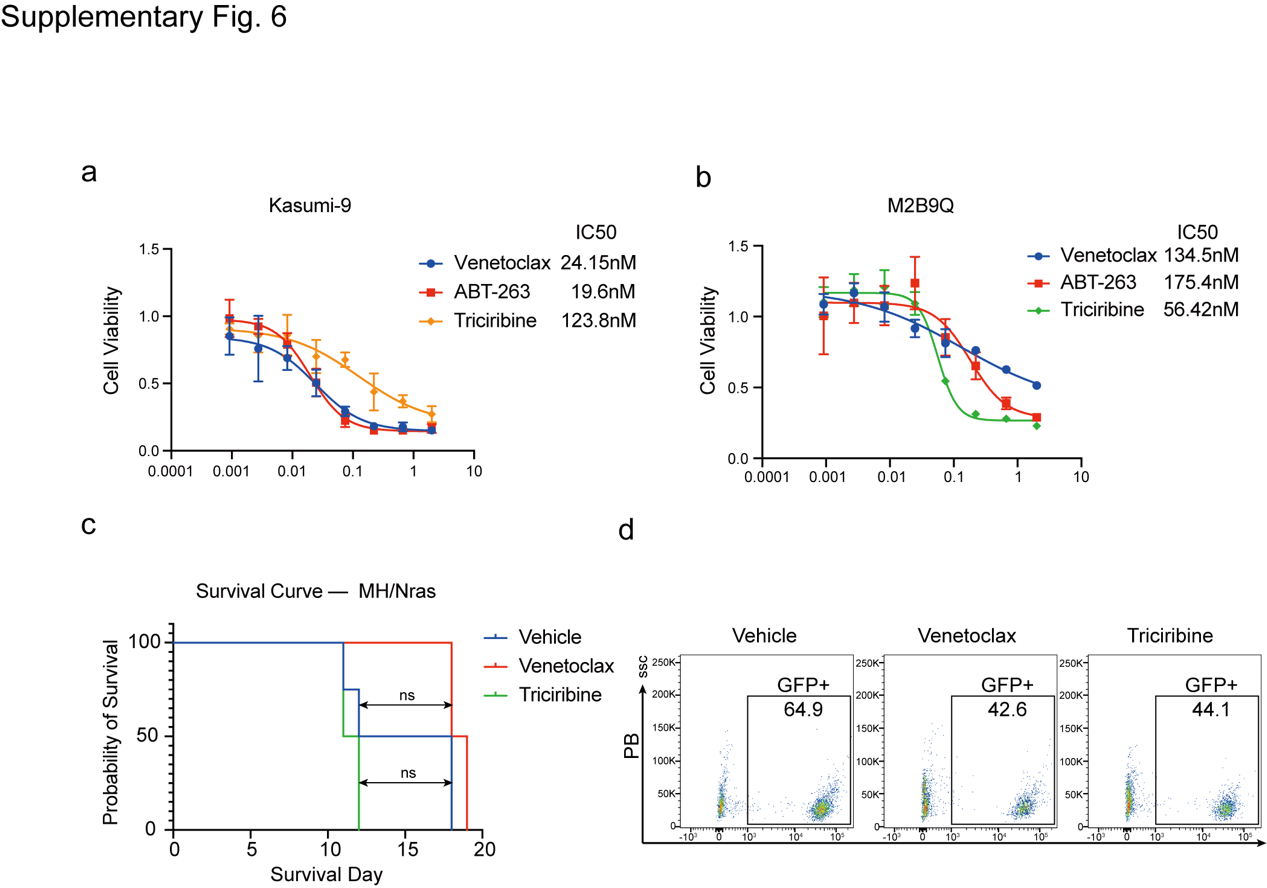
Supplementary Figure 6.** *In vivo* administration experiments with single-target inhibitors.

**a** The IC50 curves of Bcl-2 inhibitors and AKT inhibitor in Kasumi-9 cells.

**b** The IC50 curves of Bcl-2 inhibitors and AKT inhibitor in M2B9Q cells.

**c** Kaplan-Meier survival curves of *MH/NRAS^G12D^* recipient mice treated with administration of solvent (control, N=3), Venetoclax (N=3) and Triciribine (N=3). ns: not significant.

**d** Percentage of GFP-positive cells in peripheral blood from *MH/NRAS^G12D^* recipient mice treated with solvent, Venetoclax and Triciribine, respectively.

**Supplementary Methods**

**Cell cycle arrest and apoptosis assays**

For cell cycle arrest analysis, 1.5×10^6^ cells were cultured in the presence or absence of drugs as indicated for 24 hrs, harvested in chilled 70% ethanol, and stored at 4°C overnight. Subsequently, the cells were labeled with propidium iodide (Thermo Fisher Scientific) for 30 minutes at 37°C. The distribution of the cell cycle was determined using a BD LSR Fortessa-X20 and analyzed with ModFit LT 5.0.

To assess apoptosis, 1.5×10^6^ cells were cultured in the presence or absence of drugs as indicated. After incubation for 24 hrs, apoptosis and cell death were evaluated using an FITC Annexin V Apoptosis Detection Kit I (BD Pharmingen). Data acquisition was performed using a BD LSR Fortessa-X20 and analyzed with FlowJo software.

**Cell Survival Assays**

Kasumi-9 cells were cultured in 96-well plates (8×10^3^ cells/well), followed by treatment with eight-gradient concentrations of drugs for 72 hrs. Cell viability was assessed using the Cell Counting Kit-8 (Dojindo) assay. The absorbance values were measured at 450 nm using Multi-Mode Detection Platform. Each value was normalized to the readings of DMSO control. IC50 values were calculated using GraphPad Prism 9.0.

**Western blot analysis**

Cells were seeded into dishes and cultured in the presence or absence of drugs as indicated. Prior to lysis, the cells were washed with PBS. RIPA buffer (Sigma) supplemented with phosphatase inhibitors and protease inhibitors (Promega) was employed for cell lysis, while protein concentration was determined using a BCA Protein Assay Kit (Beyotime). Proteins (30 µg) were separated on Bis-Tris gels (GenScript SurePAGE), followed by transfer onto PVDF membranes (GE Healthcare). The membranes were blocked in TBST containing 5% BSA, and then incubated overnight at 4°C with primary antibodies. The membranes were rinsed with TBST and then incubated with secondary antibodies for 1 hour at RT. The results were visualized with the Chemiluminescent HRP Substrate (Millipore).

**RNA extraction and real-time PCR analysis**

The total RNA was extracted using the RNA Easy Fast Tissue/Cell Kit (TIANGEN), and cDNA synthesis was performed using the FastKing gDNA Dispelling RT SuperMix (TIANGEN). Real-time PCR was conducted employing 2× Master qPCR Mix (SYBR Green I with UDG) (Tsingke) along with specific primers.

**RNA-seq and data analysis**

Kasumi-9 cells were treated with 25nM CUDC-907 for 0h, 2h, 6h, 12h, 24h, and 48h, respectively. After harvesting, the cells were washed with PBS and RNA was extracted using the Qiagen RNA Extraction kit.

Salmon (1.4.0) was used to quantify transcript expressions directly from paired-end RNA-seq reads obtained via the Illumina NovaSeq6000 platform. The R packages DESeq2 and tximport were used to aggregate Salmon quant files, generate gene expression profiles, normalize these profiles, and conduct DEG analysis. Strict criteria for identifying DEGs were applied, specifically $\left| {log}_{2}FoldChange \right|>2$ and adjusted P<0.05. Enrichment analysis based on databases such as GO, KEGG was carried out using the R package clusterProfiler. GSEA was performed by command line tool with the input count matrix normalized. The parameter metric was set to "Signal2Noise" while other parameters were kept at default. Additional R packages used for visualization included ggplot2, gplots, ggthemes, ggrepel, and ComplexHeatmap. Gene ID conversions were completed using the R package biomaRt.

**FCM assay**

Cell suspensions from bone marrow, spleen, cell line or *ex vivo* cell model were stained as indicated antibodies, acquired using a ﻿BD LSR Fortessa-X20 and analyzed utilizing FlowJo software.

**HE staining**

Tissue samples were fixed in 4% paraformaldehyde at 4°C overnight, embedded in paraffin blocks, and sectioned to a 4-µm thickness. After complete removal of paraffin, these sections were stained with a HE staining kit (Beyotime). Images were captured via light microscopy at x10 magnification.

**IHC staining**

The formalin fixed tissues were paraffin-embedded and sliced into 4-µm slides. After deparaffinization, hydration and antigen retrieval, blocking endogenous peroxidase and nonspecific antibody binding were carried out followed by incubation with primary antibody. DAB developing with a GTVision TM III detection system/Mo&Rb (K5007, Dako) were carried out after the slides were incubated with secondary antibody labeled with HRP (GB23303, Servicebio). Slides were counterstained with hematoxylin, dehydrated with ethanol, cleaned with xylene, and mounted with resin mounting medium. Images were taken with CIC XSP-C204 microscopy. Three random fields for each sample were captured at 10× and 40× magnification. Scale bars were as indicated. Three random 40× fields were scored and positive proportions were calculated using ImageJ 1.53a and IHC Profiler.

**Surface plasmon resonance (SPR) experiments**

SPR experiments were performed on Biacore 8K (GE Healthcare) at 25℃. HDAC9 (TP315267, ORIGEN) and PI3Kα protein (Ag27546, Proteintech) was respectively immobilized on CM5 sensor chip in pH = 4.0 acetate sodium (BR100349, Cytiva) using diluted HBS-EP buffer (BR100669, Cytiva). The final response of immobilization approached 9000 RU for HDAC9 and 6000 RU for PI3Kα. The binding affinity of compound was measured using single-cycle kinetic method using diluted HBS-EP buffer with 5% DMSO. Increasing concentrations of CUDC-907 (0.014 μM, 0.041μM, 0.123 μM, 0.370 μM, 1.111 μM, 3.333 μM, 10 μM, 30 μM) were injected and flowed over the surface of sensor chip with contacting time 180 S and final dissociation time of 360 S. The response results were processed using the Biacore Insight Evaluation Software (3.0.12. 15655, GE Healthcare). The data were fit to a 1:1 Binding model.

**Molecular Docking**

To evaluate the binding energy and interaction modes between candidate drugs and their targets, we utilized AutodockVina 1.2.2, a computational protein-ligand docking software. The X-ray crystal structures of PI3K (PDB ID: 8AM0, resolution: 2.82 Å), HDAC4 (PDB ID: 2VQM, resolution: 1.80 Å), and HDAC1 (PDB ID: 4BKX, resolution: 3.00 Å) were retrieved from the Protein Data Bank (PDB). For HDAC9, its structure was predicted using AlphaFold3 based on its amino acid sequence, and the top-ranked model with the highest predicted Local Distance Difference Test (pLDDT) score was selected as the experimental structure. The chemical structure of the small molecule compound CUDC-907 was obtained from TargetMol. Proteins and ligands were prepared by converting all relevant files into PDBQT format and incorporating polar hydrogen atoms. A docking box of dimensions 40 Å × 40 Å × 40 Å with a grid spacing of 0.375 Å (0.05 nm) was defined for molecular docking analysis. To assess the affinity of candidate drugs toward their respective targets, molecular docking simulations were performed. The binding conformations and binding energies of the most favorable protein-ligand complexes were determined using AutodockVina 1.2.2. Subsequently, the protein-ligand interaction interfaces were systematically analyzed using PLIP and LigPlus, while detailed visualization of the interactions was achieved using pyMOL 2.5.
