# Supplementary material for: Molecular mechanisms of unique therapeutic potential of CUDC-907 for MEF2D fusion-driven BCP-ALL
Source: Signal Transduct Target Ther. 2025 Jul 23;10:230. doi: 10.1038/s41392-025-02310-y (PMC12283968; doi:10.1038/s41392-025-02310-y)
Supplement: Supplementary file 2 — Supplementary Table [file 41392_2025_2310_MOESM2_ESM.pdf]

Supplementary Table 1. IC50s of 22 hits.

Drug concentration(μM)	10.000	3.333	1.111	0.370	0.123	0.041	0.014	0.005	IC50(μM)	R squared
Elesclomol (STA-4783)	0.03	0.02	0.01	0.02	0.02	0.14	0.77	1.27	0.02	0.99
Elesclomol (STA-4783)	0.02	0.01	0.01	0.01	0.02	0.20	0.98	1.14		
YM155 (Sepantronium Bromide)	0.00	0.00	0.00	0.00	0.01	0.02	0.15	1.19	<0.005	
YM155 (Sepantronium Bromide)	0.00	0.00	0.00	0.00	0.01	0.02	0.07	0.37		
Bortezomib (PS-341)	0.01	0.00	0.00	0.00	0.00	0.01	0.21	0.73	0.01	0.88
Bortezomib (PS-341)	0.01	0.00	0.00	0.00	0.00	0.00	0.09	0.37		
Mitoxantrone HCl	0.01	0.01	0.01	0.01	0.03	0.14	0.21	0.21	<0.005	
Mitoxantrone HCl	0.01	0.01	0.01	0.01	0.02	0.09	0.14	0.16		
Carfilzomib (PR-171)	0.00	0.00	0.00	0.00	0.00	0.01	0.01	0.01	<0.005	
Carfilzomib (PR-171)	0.00	0.00	0.01	0.01	0.01	0.01	0.01	0.01		
Dinaciclib (SCH727965)	0.02	0.03	0.02	0.02	0.03	0.23	1.26	1.26	0.02	0.91
Dinaciclib (SCH727965)	0.01	0.02	0.02	0.02	0.02	0.06	0.50	1.19		
Triptolide (PG490)	0.01	0.02	0.02	0.03	0.02	0.03	0.27	1.00	0.01	0.97
Triptolide (PG490)	0.01	0.01	0.01	0.02	0.02	0.02	0.20	0.74		
CUDC-907	0.01	0.00	0.01	0.01	0.01	0.02	0.11	0.25	<0.005	
CUDC-907	0.01	0.00	0.01	0.01	0.01	0.04	0.13	0.01		
NSC 319726	0.02	0.05	0.02	0.01	0.01	0.01	0.02	0.05	<0.005	
NSC 319726	0.02	0.04	0.04	0.01	0.01	0.01	0.01	0.02		
Dp44mT	0.04	0.03	0.02	0.01	0.01	0.01	0.04	0.09	>10.000	
Dp44mT	0.03	0.03	0.03	0.01	0.01	0.01	0.02	0.04		
Combretastatin A4	0.00	0.00	0.00	0.01	0.03	0.15	0.36	0.40	<0.005	
Combretastatin A4	0.00	0.00	0.00	0.01	0.02	0.09	0.24	0.27		
Monomethyl auristatin E (MMAE)	0.00	0.01	0.02	0.03	0.03	0.04	0.07	0.33	<0.005	
Monomethyl auristatin E (MMAE)	0.00	0.01	0.01	0.02	0.03	0.03	0.03	0.13		
Colchicine	0.01	0.00	0.00	0.00	0.01	0.03	0.18	0.32	<0.005	
Colchicine	0.01	0.00	0.00	0.00	0.01	0.02	0.10	0.14		
Idarubicin	0.01	0.00	0.00	0.00	0.01	0.06	0.15	0.15	0.04	0.91
Idarubicin	0.01	0.00	0.00	0.00	0.00	0.03	0.10	0.08		
Podophyllotoxin	0.02	0.01	0.01	0.02	0.06	0.28	0.84	1.15	0.02	0.92
Podophyllotoxin	0.02	0.01	0.01	0.02	0.04	0.14	0.52	0.69		
Mitoxantrone	0.00	0.00	0.00	0.01	0.03	0.10	0.15	0.11	0.07	0.95
Mitoxantrone	0.01	0.00	0.00	0.00	0.03	0.14	0.18	0.15		
OTS514 hydrochloride(1338540-63-8(free base))	0.01	0.00	0.00	0.00	0.04	0.30	1.24	1.78	0.02	0.92
OTS514 hydrochloride(1338540-63-8(free base))	0.01	0.00	0.00	0.00	0.02	0.19	0.67	1.14		
Ansamitocin P-3	0.01	0.00	0.00	0.00	0.00	0.00	0.02	0.02	<0.005	
Ansamitocin P-3	0.01	0.00	0.00	0.00	0.00	0.01	0.02	0.04		
BDA-366	0.00	0.00	0.00	0.00	0.00	0.01	0.06	0.12	<0.005	
BDA-366	0.00	0.00	0.00	0.00	0.00	0.01	0.04	0.06		
1S,3R-RSL3	0.00	0.01	0.01	0.10	0.65	1.01	1.04	0.84	0.12	0.94
1S,3R-RSL3	0.00	0.00	0.01	0.05	0.43	1.35	1.22	0.91		
VLX1570	0.01	0.10	0.66	0.96	1.17	1.40	1.28	0.73	1.39	0.87
VLX1570	0.01	0.13	0.87	1.32	1.35	1.44	1.32	0.87		
Daunorubicin HCl	0.01	0.01	0.01	0.01	0.01	0.06	0.18	0.17	0.04	0.90
Daunorubicin HCl	0.01	0.01	0.01	0.01	0.01	0.05	0.13	0.09		

Supplementary Table 2. IC50s of CUDC-907 in BCP-ALL Cell Lines.

Drug concentration(μM)	10.000	3.333	1.111	0.370	0.123	0.041	0.014	0.005	IC50(μM)	R squared
JM1	0.03	0.03	0.02	0.79	1.02	1.02	1.03	1.02	0.5303	0.9907
	0.03	0.03	0.03	1.05	1.05	1.05	1.05	1.04		
	0.05	0.06	0.08	1.05	1.05	1.03	1.03	1.06		
Nalm6	0.28	0.29	0.40	0.68	0.84	0.87	0.84	0.83	0.6001	0.9857
	0.29	0.34	0.44	0.70	0.80	0.88	0.87	0.87		
	0.28	0.32	0.41	0.75	0.89	0.81	0.89	0.94		
RCH-ACV	0.06	0.07	0.07	0.90	0.90	0.99	0.95	1.01	0.4867	0.9758
	0.06	0.06	0.07	0.75	0.81	0.89	0.95	0.94		
	0.07	0.07	0.08	0.60	0.83	0.94	1.04	0.99		
REH	0.05	0.05	0.20	0.84	0.83	0.85	0.88	0.86	0.8587	0.9874
	0.06	0.05	0.20	0.88	0.93	0.91	0.83	0.91		
	0.06	0.06	0.26	0.98	0.99	0.99	0.94	0.94		
SEM	0.09	0.08	0.08	0.73	0.89	0.90	0.92	0.93	0.4654	0.9715
	0.08	0.08	0.08	0.69	0.92	1.09	0.81	1.01		
	0.09	0.08	0.08	0.86	0.91	0.99	1.17	0.99		
Sup-B15	0.07	0.08	0.08	0.65	1.02	1.02	1.08	1.31	0.4532	0.9619
	0.08	0.08	0.08	0.90	1.03	1.17	0.99	0.95		
	0.08	0.07	0.07	0.83	0.88	0.98	0.97	0.90		

Drug concentration(μM)	2.000	0.667	0.222	0.074	0.025	0.008	0.003	0.001	IC50(μM)	R squared
Kasumi-9	0.15	0.14	0.14	0.14	0.23	0.49	0.67	0.86	0.0113	0.9421
	0.10	0.10	0.10	0.10	0.28	0.63	0.98	1.05		
	0.10	0.10	0.10	0.10	0.28	0.63	0.98	1.05		

Drug concentration（μM）	0.2000	0.0667	0.0222	0.0074	0.0025	0.0008	0.0003	0.0001	IC50(μM)	R squared
M2B9Q	0.05	0.13	0.37	0.68	0.94	0.92	0.96	1.00	0.0145	0.9918
	0.05	0.15	0.37	0.75	1.00	0.99	1.01	0.97		
	0.05	0.15	0.37	0.79	0.93	1.03	1.01	0.99		

Supplementary Table 3. IC50s of CUDC-907 in bone marrow samples and PDX cells from primary patients.

Drug concentration(nM)	0.032	0.160	0.800	4.000	20.000	100.000	IC50(nM)	R squared
PT-1 (MH)	101.3552	96.51852	63.71516	29.35736	17.26671	13.97125	1.189	0.9887
	105.5879	96.71683	81.97151	29.23607	17.08167	16.34153		
	110.8132	103.7386	71.70737	34.91446	20.41576	16.80412		
PT-2 (MS)	96.03777	96.4976	90.32018	40.5059	33.68669	38.26515	2.035	0.9565
	110.6421	108.1783	104.9038	43.94573	36.44903	35.53414		
	115.9131	116.5265	108.083	60.31707	38.63061	41.63146		
PT-3 (MB)	89.79176	89.11832	58.8057	3.295592	3.354852	4.030944	0.9486	0.9944
	98.58962	89.5523	59.52738	3.464104	4.225217	3.857706		
	102.267	89.93962	61.05894	3.87395	4.337572	5.378552		

Drug concentration(nM)	0.032	0.160	0.800	4.000	20.000	100.000	IC50(nM)	R squared
PDX-1 (MB)	104.2401	89.44943	95.80492	60.89849	1.327268	3.984475	4.655	0.9818
	98.21274	96.32242	83.03085	52.11163	1.09009	1.191291		
	111.1906	98.58444	86.55333	57.15069	1.296122	1.269606		
PDX-2 (MS)	108.8722	117.3174	104.6966	30.89639	1.458111	3.982017	2.637	0.9814
		127.8462	122.5355	37.51136	1.691427	5.61788		
	136.4527	137.6091	127.6378	40.50781	1.809882	5.969586		
PDX-3 (MB)	97.42723	92.68579	88.63801	59.0928	5.370464	16.88997	4.081	0.986
	101.9654	95.38315	98.4886	59.3205	5.509366	16.89149		
	91.64728	94.72466	89.0104	57.80826	5.227511	16.75548		
PDX-4 (MH)	105.785	105.5859	84.92776	3.317582	1.419259	1.542035	1.272	0.9981
	100.693	102.4639	87.18357	2.788011	1.349389	1.495681		
	104.2282	95.50861	87.63363	2.68776	1.303657	1.4694		

Number	Age	Sex	Fusion Type
PT-1	15	Female	*MEF2D::HNRNPUL1*
PT-2	14	Female	*MEF2D::SS18*
PT-3	6	Male	*MEF2D::BCL9*
PDX-1	11	Male	*MEF2D::BCL9*
PDX-2	4	Female	*MEF2D::SS18*
PDX-3	70	Female	*MEF2D::BCL9*
PDX-4	68	Female	*MEF2D::HNRNPUL1*

Supplementary Table 4. List of antibodies used for western blot analysis in this paper.

Targets	Supplier	Catalog number	Dilution	Isotype	Usage
PI3 Kinase p110α	CST	4249	1:1000	Rabbit IgG	WB
MEF2D	proteintech	14353-1-AP	1:1000	Rabbit IgG	WB
DYRK1A	CST	8765	1:1000	Rabbit IgG	WB
Btk	CST	8547	1:1000	Rabbit IgG	WB
p-Btk	CST	87457	1:1000	Rabbit IgG	WB
CREB	CST	9197	1:1000	Rabbit IgG	WB
p-CREB	CST	9198	1:1000	Rabbit IgG	WB
Akt	CST	4685	1:1000	Rabbit IgG	WB
p-AKT	CST	4060	1:1000	Rabbit IgG	WB
p44/42 MAP Kinase	CST	4695	1:1000	Rabbit IgG	WB
p-p44/42 MAPK	CST	4379	1:1000	Rabbit IgG	WB
FoxO1	CST	2880	1:1000	Rabbit IgG	WB
Pro-Apoptosis Bcl-2 Family Antibody Sampler Kit II	CST	98322	1:1000	Rabbit IgG	WB
Pro-Survival Bcl-2 Family Antibody Sampler Kit II	CST	17229	1:1000	Rabbit IgG	WB
Anti-rabbit IgG, HRP-linked Antibody	CST	7074	1:10000	Goat	WB
Anti-mouse IgG, HRP-linked Antibody	CST	7076	1:10000	Horse	WB
β-Actin Antibody (C4)	santa cruz	sc-47778	1:1000	mouse IgG_1	WB
Acetyl-Histone H3 Antibody Sampler Kit	CST	9927	1:1000	Rabbit IgG	WB
PARP	CST	9532	1:1000	Rabbit IgG	WB
c-Myc	abcam	ab32072	1:1000	Rabbit IgG	WB
Caspase-3	CST	9662	1:1000	Rabbit IgG	WB
Caspase-9	CST	9508T	1:1000	mouse IgG_1	WB
Phospho-FoxO1	CST	9461T	1:1000	Rabbit IgG	WB
Phospho-c-Myc	CST	13748	1:1000	Rabbit IgG	WB
Histone H3	abcam	ab32356	1:1000	Rabbit IgG	WB

Supplementary Table 5. List of antibodies used in the flow cytometric analysis in this paper.

Antibody	Clone or Composition	Species	Product Number	Supplier	Fluor
B220	RA3-6B2	mouse	561101	BD Pharmingen	PerCP-Cyanine5.5
CD19	1D3	mouse	47-0193-80	eBioscience™	APC-eFluor 780
CD19	SJ25C1	human	562947	BD Pharmingen	BV510
CD179b (lambda5)	HSL11	human	349804	Biolegend	PE
pre-BCR	HSL2	human	347904	Biolegend	PE
Igμ	SA-DA4	human	17-9998-42	eBioscience™	APC
IgD	IA6-2	human	11-9868-42	eBioscience™	FITC
BAFF Receptor	11C1	human	558097	BD Pharmingen	PE
BCMA/TNFRSF17	Clone # 1004023	human	FAB1931G	R&D Systems	Alexa Fluor 488
FITC Annexin V Apoptosis Detection Kit I			556547	BD Pharmingen	

Supplementary Table 6.List of antibodies used for HE and IHC staining in this paper.

Targets	Supplier	Catalog number	Dilution	Usage
HDCA1	Servicebio	GB11333	1:300	IHC
CD19	Servicebio	GB11061-1	1:200	IHC
PI3KCA	Servicebio	GB11769	1:500	IHC
BCL2	Servicebio	GB114830	1:500	IHC
HRP conjugated Goat Anti-Rabbit IgG	Servicebio	GB23303	1:200	IHC

Supplementary Table 7. List of Primers used for qRT-PCR in this paper.

	Primer name	Sequence	Product length
1	BLNK-F<br>BLNK-R	CCCGCCAGTCAGAAGTTGAG<br>AGTCCCTTCGAGGAACACTTG	127 bp
2	MYC-F<br>MYC-R	GGCTCCTGGCAAAAGGTCA<br>CTGCGTAGTTGTGCTGATGT	119 bp
3	IRF4-F<br>IRF4-R	GCTGATCGACCAGATCGACAG<br>CGGTTGTAGTCCTGCTTGC	111 bp
4	CXCR4-F<br>CXCR4-R	ACTACACCGAGGAAATGGGCT<br>CCCACAATGCCAGTTAAGAAGA	133 bp
5	CCND3-F<br>CCND3-R	TACCCGCCATCCATGATCG<br>AGGCAGTCCACTTCAGTGC	128 bp
6	RAG1-F<br>RAG1-R	CTGTTCCGGGTGAGATCCTTT<br>TAACAATGGCTGAGTTGGGAC	150 bp
7	GAPDH-hum-F<br>GAPDH-hum-R	TGTTGCCATCAATGACCCCTT<br>CTCCACGACGTACTCAGCG	202 bp
8	DYRK1A-F<br>DYRK1A-R	AAGAAGCGAAGACACCAACAG<br>TTTCGTAACGATCCATCCACTTT	139 bp
9	HDAC1-F<br>HDAC1-R	CTACTACGACGGGGATGTTGG<br>GAGTCATGCGGATTCGGTGAG	77 bp
10	HDAC2-F<br>HDAC2-R	ATGGCGTACAGTCAAGGAGG<br>TGCGGATTCTATGAGGCTTCA	112 bp
11	HDAC3-F<br>HDAC3-R	TCTGGCTTCTGCTATGTCAACG<br>CCCGGTCAGTGAGGTAGAAAG	136 bp
12	HDAC4-F<br>HDAC4-R	CCTGGGAATGTACGACGCC<br>CCCGTCTTTCCTGCGTAAC	136 bp
13	HDAC5-F<br>HDAC5-R	TCTTGTCGAAGTCAAAGGAGC<br>GAGGGGAACTCTGGTCCAAAG	108 bp
14	HDAC6-F<br>HDAC6-R	ACCCCAGTGTCCTCTATTTCTC<br>CCTGGTTCCAAGGCACATTGA	135 bp
15	HDAC8-F<br>HDAC8-R	TCGCTGGTCCCGGTTTATATC<br>TACTGGCCCGTTTGGGGAT	82 bp
16	HDAC9-F<br>HDAC9-R	AGTAGAGAGGCATCGCAGAGA<br>GGAGTGTCTTTCGTTGCTGAT	141 bp
