# Supplementary figures and images for: Molecular mechanisms of unique therapeutic potential of CUDC-907 for MEF2D fusion-driven BCP-ALL
Source: Signal Transduct Target Ther. 2025 Jul 23;10:230. doi: 10.1038/s41392-025-02310-y (PMC12283968; doi:10.1038/s41392-025-02310-y)

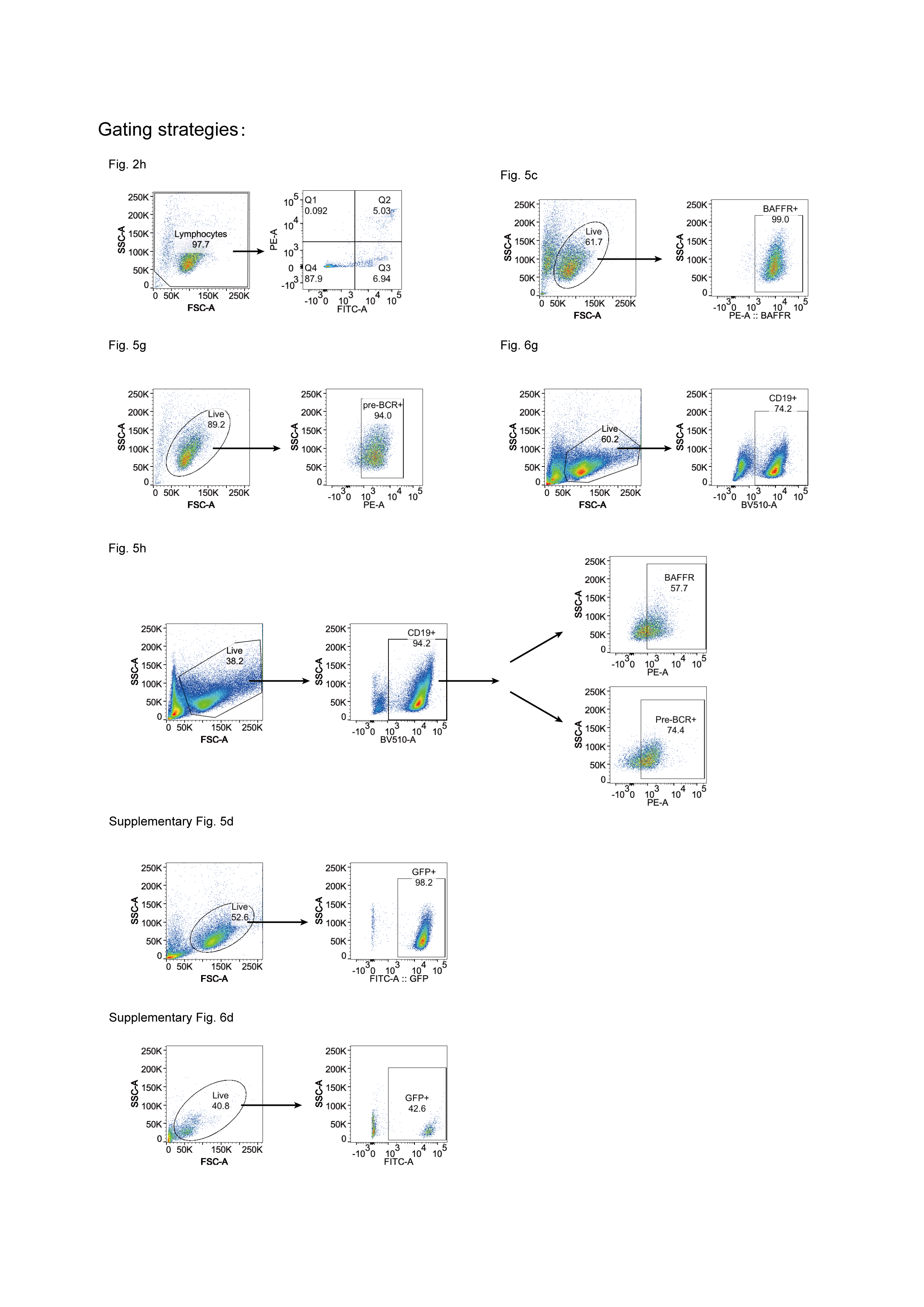

Supplement: Supplementary file 3 — Gating strategies [file 41392_2025_2310_MOESM3_ESM.tif]

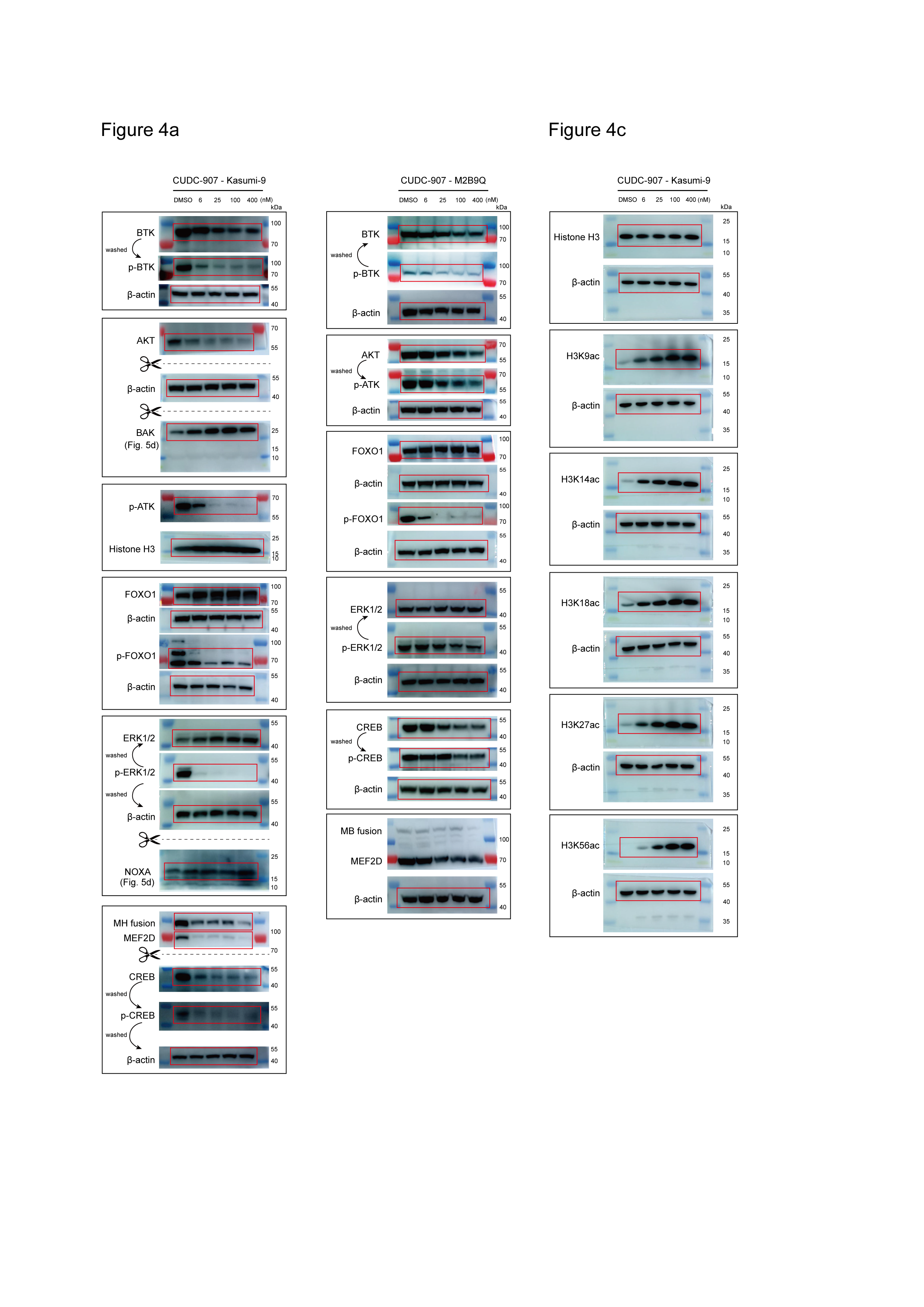

Supplement: Supplementary file 4 — Uncropped blots-1 [file 41392_2025_2310_MOESM4_ESM.tif]

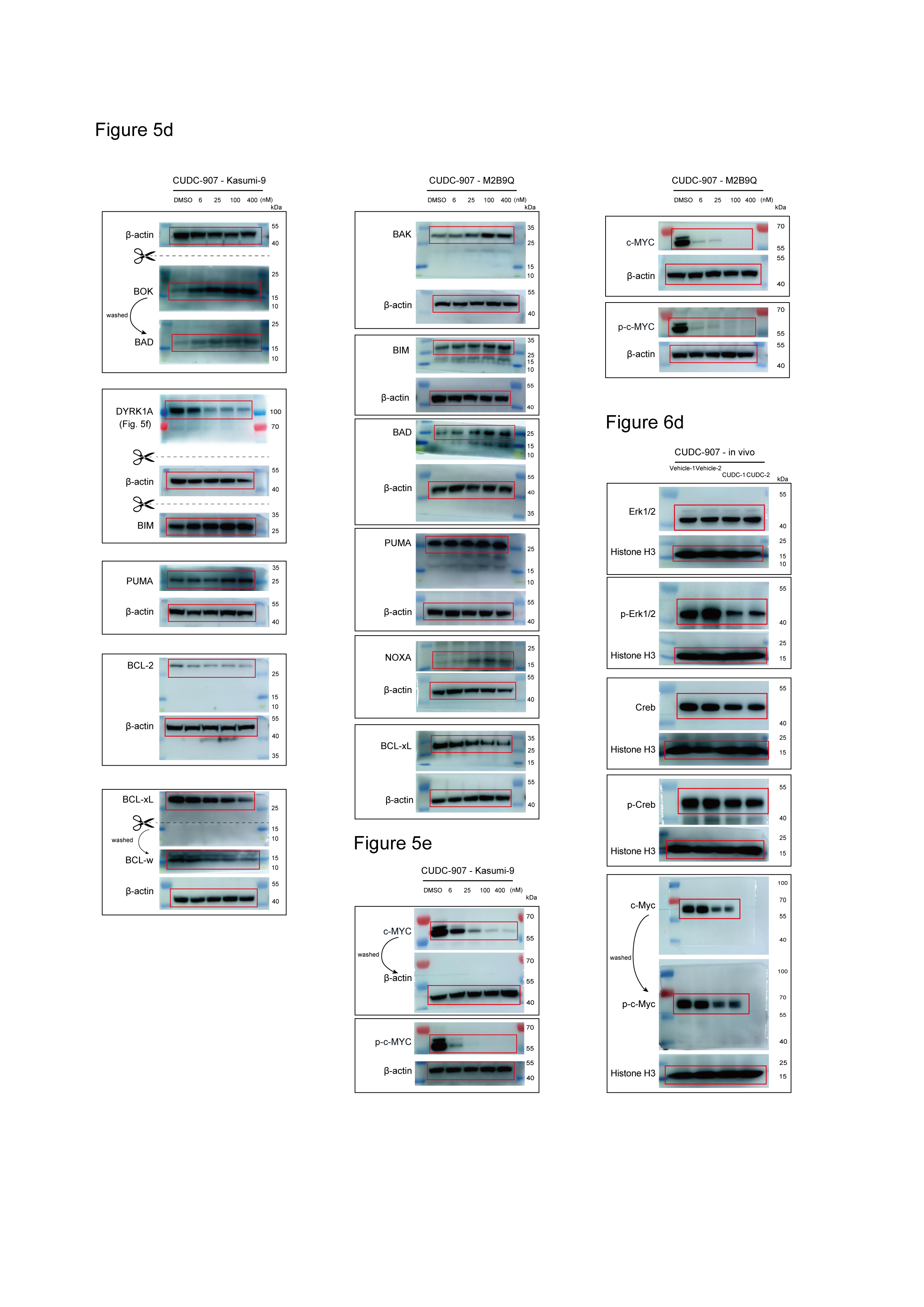

Supplement: Supplementary file 5 — Uncropped blots-2 [file 41392_2025_2310_MOESM5_ESM.tif]

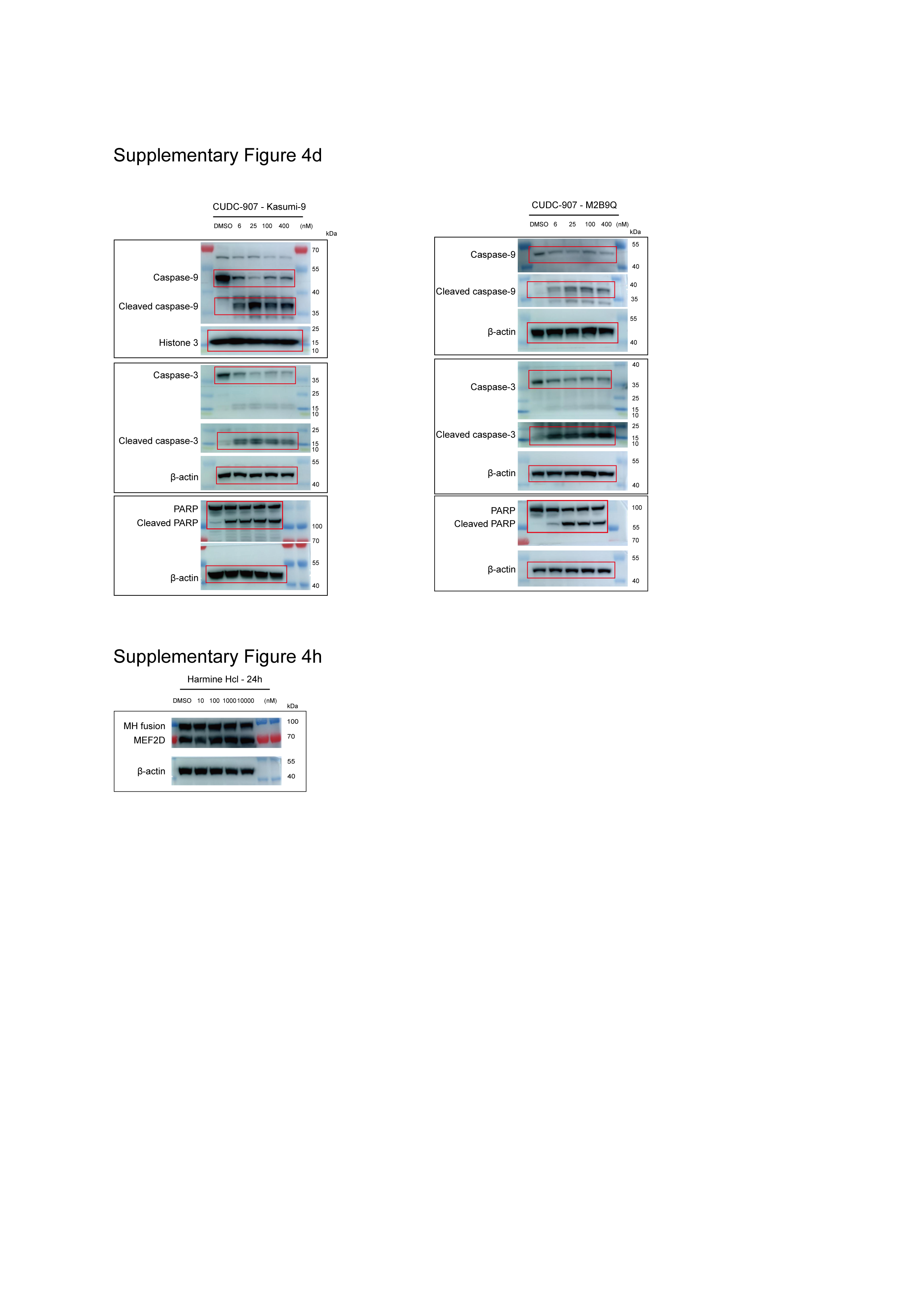

Supplement: Supplementary file 6 — Uncropped blots-3 [file 41392_2025_2310_MOESM6_ESM.tif]
